# Comparing efficacy of neoadjuvant therapy of triple-negative breast cancer: A Bayesian network meta-regression analysis

**DOI:** 10.1097/MD.0000000000046962

**Published:** 2026-01-09

**Authors:** Yicheng Jiang, Jiajia Zeng, Jian Liu, Wenbo Deng, Ulf Dietrich Kahlert, Ruijun Tang, Meng Xu, Wenjie Shi, Qiang Wang

**Affiliations:** a Department of Breast Surgery, Guilin Municipal Hospital of Traditional Chinese Medicine, Guilin, China; b Department of Medical Aesthetic Surgery, The First Affiliated Hospital of Guilin Medical University, Guilin, China; c Molecular and Experimental Surgery, University Clinic for General-, Visceral-, Vascular- and Trans-Plantation Surgery, Medical Faculty University Hospital Magdeburg, Otto-von Guericke University, Magdeburg, Germany.

**Keywords:** Bayesian network meta-analysis, chemotherapy, immune checkpoint inhibitor, neoadjuvant treatment, network meta-regression analysis, triple-negative breast cancer

## Abstract

**Background:**

Triple-negative breast cancer (TNBC) is a subtype of breast cancer with a poor prognosis and limited treatment options. Currently, nonmetastatic TNBC is mostly treated with neoadjuvant chemotherapy, but comparisons between these neoadjuvant regimens are dearth.

**Methods:**

PubMed, Embase, Medline, Cochrane Library, Web of ClinicalTrials.gov, and major international conference databases were systematically searched for randomized controlled trials (RCTs) on the efficacy of various neoadjuvant chemotherapy treatments in patients with TNBC from inception to January 2025. The primary research endpoint was the pathological complete response (pCR) rate. The secondary endpoint was the odds ratios (ORs) at different time points of event-free survival (EFS) and overall survival (OS). The tertiary endpoints were the hazard ratios (HRs) of EFS and OS compared by Bayesian network meta-analysis, as well as corresponding Bayesian network meta-regression analysis with the median follow-up time as the covariate. The above processes were conducted by RStudio 4.2.2 orchestrated with STATA 17.0 MP.

**Results:**

For the primary endpoint, compared to regimens containing anthracycline and taxanes (AT), regimens containing anthracycline, taxanes, platinum, and programmed cell death protein-1 (ATPtPD1) showed significant higher pCR rate (OR = 5.68). For the secondary endpoint, compared to AT, ATPtPD1 showed significant longer EFS/OS. For EFS: OR = 2.28 at 18th month; OR = 2.43 at 24th month; OR = 3.21 at 30th month; OR = 4.23 at 36th month; OR = 4.62 at 42nd month; OR = 4.04 at 48th month. For OS: OR = 3.56 at 18th month; OR = 2.23 at 24th month; OR = 2.49 at 30th month; OR = 2.49 at 36th month; OR = 3.17 at 42nd month; OR = 2.97 at 48th month. For the tertiary endpoints, for HR of EFS, compared to AT, ATPtPD1 indicated significant advantage (HR = 2.24, 95% confidence interval [CI]: 1.42–3.59), after meta-regression analysis, shows advantages as well (HR = 2.29, 95% CI: 1.39–3.89). For HR of OS, compared to AT, ATPtPD1 indicated significant advantage (HR = 2.67, 95% CI: 1.03–7.35), after meta-regression analysis, shows advantages as well (HR = 2.70, 95% CI: 1.18–6.33).

**Conclusions:**

Considering efficacy on pCR and OS/EFS together, ATPtPD1 should be considered as the best recommendation in neoadjuvant therapies of TNBC.

## 1. Introduction

Breast cancer is the most common malignant tumor among women in the world, and it is also the second leading cause of cancer death in women. During 2012 to 2021, the incidence rate of breast cancer increased by 1% every year.^[1]^ It poses a great threat to the physical and mental health of patients worldwide.

The molecular subtypes of breast cancer also vary according to race and ethnicity. White women have the highest proportion of hormone receptor positive/human epidermal growth factor receptor 2 negative breast cancer, while Black women have a significantly higher proportion of triple-negative breast cancer (TNBC) than other groups.^[1]^

TNBC accounts for 12% to 17% of all breast cancers.^[2]^ Compared to other subtypes of breast cancer, TNBC patients suffer worse clinical outcomes.^[2,3]^ TNBC patients who achieve pathological complete response (pCR) will have a better prognosis than non-pCR.^[4]^ In early stage TNBC patients, neoadjuvant chemotherapy has become a standard approach and is more likely to achieve pCR than non-TNBC patients.^[4]^

Neoadjuvant chemotherapy is a treatment approach administered before primary surgery. It has revolutionized the management of breast cancer, particularly in aggressive subtypes, such as TNBC. Its primary goal is to shrink tumors, making them more amenable to conservative surgical procedures and reducing the presence of early micrometastases.^[5]^ Accordingly, regulatory guidance supports the use of the pCR as an end point for clinical testing of neoadjuvant treatment in patients with early TNBC.^[6,7]^

Anthracyclines, cyclophosphamides, and taxanes are the essential drugs in neoadjuvant chemotherapy regimens for TNBC.^[8]^ As the higher pCR rate and event-free survival (EFS)/overall survival (OS) benefit, platinum plays an important antitumor roles in neoadjuvant chemotherapy for TNBC.^[9,10]^ Platinum is a DNA cross-linking agent, which can cross-connect with the DNA after entering the tumor cells, cause DNA strand breaks in tumor cells.^[11]^

Currently, poly-ADP-ribose polymerase inhibitors (PARPi) are used in neoadjuvant chemotherapy for TNBC patients, especially with BRCA gene mutations.^[12]^ PARPi are a class of drugs that target the DNA repair mechanism of tumor cells. Their core mechanism of action is based on the principle of “synthetic lethality,” which selectively kills cancer cells by interfering with the DNA repair process.^[13]^

The vascular endothelial growth factor, such as bevacizumab, is an important regulator of tumor angiogenesis and metastasis.^[14,15]^ Bevacizumab plays various roles in the tumor blood vessels by specifically binding to vascular endothelial growth factor and blocking its interaction with receptors.^[16]^

In recent years, immune checkpoint inhibitor (ICI) therapy has been successful in metastatic TNBC.^[17]^ ICI therapy is directed against the interaction between the programmed death 1 (PD-1) and programmed death ligand 1 (PD-L1).^[18]^ PD-1 is a co-inhibitory molecule expressed by activated T-cells when antigen-presenting cells or tumor cells are combined with PD-L1, which further lead to inhibiting the T-cell activation and suppressing the body’s antitumor immune response.^[19]^

Chemotherapy schemes composed of various chemotherapeutic drugs were used in neoadjuvant chemotherapy of TNBC. Some obtained high pCR rates. However, the improvement in pCR did not translate into benefits for EFS or OS.

In recent years, there have been an increasing number of Bayesian network meta-analyses (NMAs) on neoadjuvant chemotherapy regimens for TNBC, with nonconclusive evidence. In 2022, Li et al^[20]^ published an NMA evaluating 8 neoadjuvant treatment options for TNBC. The treatment regimen included the combination of platinum, bevacizumab, PARPi, and ICI. In this previous study, the observation indicator was pCR.^[20]^ Similarly, in 2024, Liu et al^[21]^ showed that bevacizumab associated with platinum-containing regimens is likely to be the optimal treatment option for neoadjuvant TNBC, but this results are limited. In terms of data analytics, they only considered the covariates of hazard ratio (HR) and inferred the result by surface under the cumulative ranking curve (SUCRA), making the final results less reliable.^[21]^

Hence, we conducted this Bayesian NMA of randomized controlled trials (RCTs) about neoadjuvant therapy for TNBC, which includes direct and indirect comparisons among regimens, to identify the most effective maintenance treatment for TNBC patients that, in turn, could improve the oncological clinical practice.

## 2. Methods

This Bayesian NMA was guided by the PRISMA guideline (Preferred Reporting Items for Systematic Reviews and Meta-analysis).^[22]^

### 2.1. Search strategy

We searched Google Scholars, Cochrane Library (CENTRAL), PubMed, Scopus, Embase, Web of ClinicalTrials.gov, and major international conference databases from inception to January 2025, with the following Mesh terms: (“Breast Cancer” OR “Breast Carcinoma” OR “Breast Neoplasm” OR “Breast Tumor” OR “Breast Malignant Tumor”) AND (“Neoadjuvant” OR “Neoadjuvant therapy” OR “Neoadjuvant Treatment”) AND (“Triple Negative” OR “ER Negative PR Negative HER2 Negative” OR “Endocrine Negative HER2 Negative”) AND (“Randomized” OR “Randomization” OR “Allocation Random”).

### 2.2. Selection criteria

The inclusion criteria were as follows: the study subjects were diagnosed TNBC patients without metastatic; the study design was RCT about neoadjuvant therapy; sufficient information was provided on pCR and/or PFS and/or OS.

The exclusion criteria were as follows: the data required for analysis was not reported; articles were observational studies, letters, or review; articles reported by non-English literature.

### 2.3. Data extraction and quality assessment

Two investigators independently searched and assessed the eligibility of each study by reading the title and abstract or even full-text when necessary. Data were independently extracted by them also. Any discrepancy was arbitrated by the senior reviewer. At last, the risk of bias for each included RCT was assessed by Cochrane Risk of Bias tool. The following information were collected: names of the first authors, publication year, country, number of patients, condition, therapeutic drugs, treatment dosage, and the outcomes included numbers of patients reached pCR, HRs, and confidence intervals (CIs) associated with EFS and OS. Subsequently, the data regarding EFS and OS at 18, 24, 30, 36, 42, and 48 months were harvested from Kaplan–Meier curve by GetData 2.26 (https://getdata.sourceforge.net/download).

### 2.4. Research endpoint

The primary research endpoint was the pCR rate. The secondary endpoint was the odds ratios (ORs) at different time points of EFS and OS. The tertiary endpoints were the HRs of EFS and OS compared by Bayesian NMA, as well as corresponding Bayesian network meta-regression analysis with the median follow-up time as the covariate.

### 2.5. Data analysis

For pCR and EFS/OS rate at each time node, ORs were generated by NMA using STATA 17.0 MP (https://www.stata.com/statamp/) to make pairwise comparisons among regimens. SUCRA was also formulated; a higher SUCRA indicates a higher probability of being the better treatment. However, whether the effect size between any pair with corresponding SUCRAs reached the significance was determined by net-league table, also called matrix in algebra. Inconsistency and consistency tests were performed to examine the existence of inconsistency. Publication bias was assessed by funnel plot as well.

For HRs regarding OS and EFS in each study Napierian logarithm HR (lnHR) and standard error of lnHR (selnHR) for each study were calculated by STATA 17.0 MP. Subsequently these data (lnHR and selnHR for OS and PFS) were input into RStudio 4.2.2 (https://cran.rstudio.com/bin/windows/base) by “gemtc” package to conduct Bayesian NMA to generate pairwise HRs, SUCRA, and matrix. Markov chain Monte Carlo was used to obtain posterior distributions, with 2000 burn-ins and 300,000 iterations of 4 each chain and a thinning interval of 10 for each outcome. Brooks–Gelman–Rubin diagnostics and Trace and density plots were used to evaluate and visualize the convergence of the model over iterations. For heterogeneity analysis, if *I*^2^ < 50% and *P* > .01, fixed effect model would be implemented; if 50% < *I*^2^ < 75%, random effect model would be carried out; if *I*^2^ > 75%, Galbraith plot would be drawn to preclude the studies outside the outlines.

Finally, in the sensitivity analysis, we used median follow up time as a covariate to perform meta-regression analyses to eliminate potential confounding factors.

If the 95% CI of the comparison value is greater than or <1, it indicates a significant difference in the comparison result.

## 3. Results

### 3.1. Characteristics of the included studies

**Figure 1.**
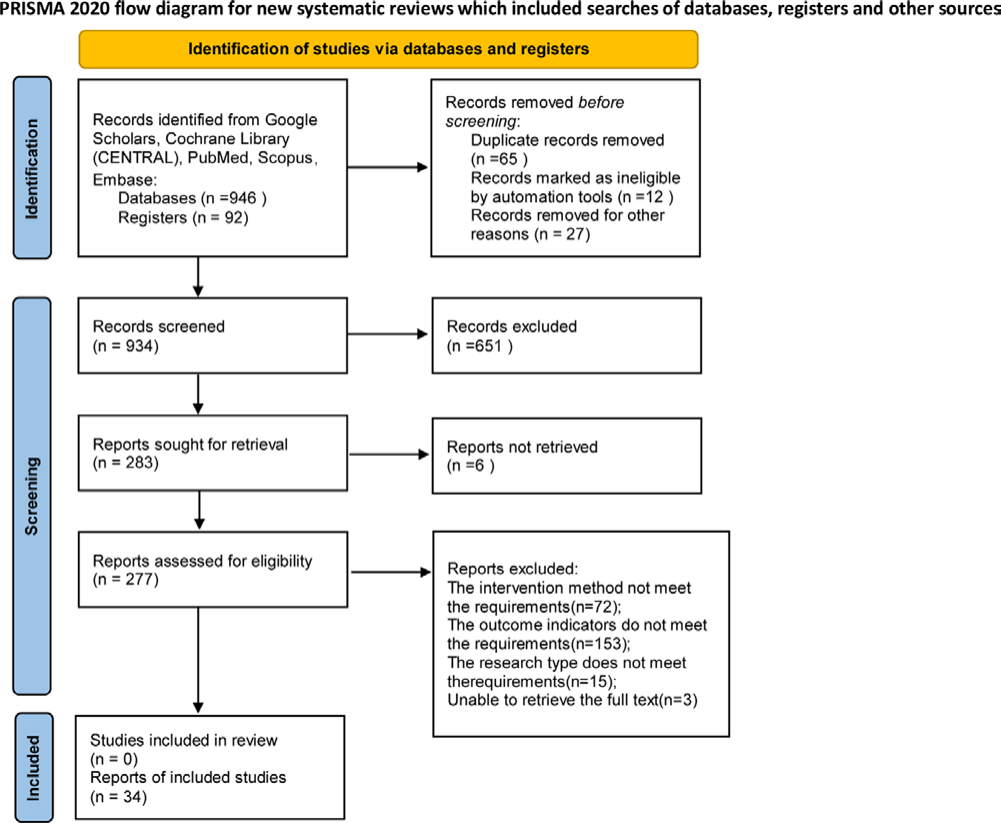
Preferred Reporting Items for Systematic Reviews and Meta‑analyses flowchart illustrating the selection of studies included in the present study.

A detailed description of the included studies can be found in Table 1. Initially, we retrieved 1038 articles from all the 5 databases; after meticulous screening, only 34 studies included 25 RCTs reporting 6838 patients were eligible for our study (Fig. 1).^[9,10,12,23–53]^ Seventeen different maintenance regimes were evaluated within the included studies, namely, containing anthracyclines and taxanes (AT); containing anthracyclines, taxanes, and bevacizumab (ATBev); containing anthracyclines, taxanes, and everolimus (ATEve); containing anthracyclines, taxanes, and gemcitabine (ATGem); containing anthracyclines, taxanes, and PD-1 (ATPD1); containing anthracyclines, taxanes, and PD-L1 (ATPDL1); containing anthracyclines, taxanes, and platinum (ATPt); containing anthracyclines, taxanes, platinum, and bevacizumab (ATPtBev); containing anthracyclines, taxanes, platinum, and PARPi (ATPtPARPi); containing anthracyclines, taxanes, platinum, and PD-1 (ATPtPD1); containing anthracyclines, taxanes, platinum, and PD-L1 (ATPtPDL1); containing anthracyclines, taxanes, and capacitabine (ATX); containing taxanes only (T); containing taxanes and PARPi (TPARPi); containing taxanes and platinum (TPt); containing taxanes, platinum, and everolimus (TPtEve); containing taxanes, platinum, and PD-L1 (TPtPDL1). Patients included come from Asia, Europe, South/North America, and Africa. As shown in Figure S1, Supplemental Digital Content, https://links.lww.com/MD/R101, the risk of bias assessment conducted showed that there was no high risk of bias in the included studies.

**Table 1:** Characteristics of neoadjuvant therapy of triple-negative breast cancer studies included in the Bayesian network meta-analysis.

Trial	Author	Year	Country	Treatment	Sample size	Dosage	Outcomes
BrighTNess^[23,24]^	Loibl	2018, 2021	Germany	Paclitaxel + doxorubicin + cyclophosphamide; paclitaxel + carboplatin + doxorubicin + cyclophosphamide; paclitaxel + carboplatin + veliparib + doxorubicin + cyclophosphamide	158; 160; 316	Paclitaxel 80 mg/m^2^ weekly for 12 doses, carboplatin AUC6 iv. every 3 weeks for 4 cycles, veliparib [50 mg orally, twice a day]; doxorubicin [60 mg/m^2^] and cyclophosphamide [600 mg/m^2^] every 2 or 3 weeks for 4 cycles.	pCR rate; EFS HR and curve; OS HR and curve
ChiCTR-TRC-14005019^[9,25]^	Wu Yan	2018 2022	China	Docetaxel + epirubicin + lobaplatin; Docetaxel + epirubicin	101; 99	Epirubicin 80 mg/m^2^, docetaxel 75 mg/m^2^, lobaplatin 30 mg/m^2^	pCR rate; EFS (DFS) HR and curve; OS HR and curve
NCT00242203^[26]^	Jovanović	2017	USA	Paclitaxel + cisplatin + everolimus; paclitaxel + cisplatin	96; 49	Everolimus 30 mg weekly or the equivalent 5 mg daily, paclitaxel 80 mg/m^2^ weekly, cisplatin 25 mg/m^2^ weekly	EFS HR and curve
GEICAM/2006-03^[27]^	Alba	2012	Spain	Docetaxel + epirubicin + cyclophosphamide + carboplatin; docetaxel + epirubicin + cyclophosphamide	48; 46	Epirubicin 90 mg/m^2^, cyclophosphamide 600 mg/m^2^ iv on day 1 every 21 days for 4 cycles, docetaxel 100 mg/m^2^ administered iv, carboplatin AUC6 administered iv on day 1 every 21 days for 4 cycles	pCR rate
IMpassion031^[28]^	Mittendorf	2020	USA	Nab-paclitaxel + nab-paclitaxel + cyclophosphamide + atezolizumab; nab-paclitaxel + nab-paclitaxel + cyclophosphamide	165; 168	Nab-paclitaxel 125 mg/m^2^ every week for 12 weeks, doxorubicin 60 mg/m^2^, cyclophosphamide 600 mg/m^2^ every 2 weeks for 8 weeks, intravenous atezolizumab 840 mg every 2 weeks	pCR rate; EFS HR; OS HR
NCI 10013^[29]^	Ademuyiwa	2021	USA	Paclitaxel + carboplatin + atezolizumab; paclitaxel + carboplatin	45; 16	Carboplatin AUC5 every 3 weeks × 4 cycles, paclitaxel 80 mg/m^2^ every week × 12 weeks, atezolizumab 1200 mg every 3 weeks × 4 cycles	pCR rate
NCT01276769^[30]^	Zhang	2016	China	Paclitaxel + carboplatin; paclitaxel + epirubicin	47; 44	Paclitaxel [175 mg/m^2^, day 1], carboplatin [AUC5, day 2], epirubicin [75 mg/m^2^, day 1] every 3 weeks for 4 to 6 cycles	pCR rate; EFS (RFS) curve; OS curve
WSG-ADAPT-TN^[31,32]^	Gluz	2018; 2022	Germany	Nab-paclitaxel + gemcitabine + epirubicin + cyclophosphamide; nab-paclitaxel + carboplatin + epirubicin + cyclophosphamide	178; 146	Nab-paclitaxel 125 mg/m^2^, gemcitabine 1000 mg/m^2^ days 1, 8, carboplatin AUC2 day 1, 8 q3w. Epirubicin 90 mg/m^2^, cyclophosphamide 600 mg/m^2^ iv on day 1 every 21 days for 4 cycles	pCR rate; EFS (DFS) HR and curve; OS HR and curve
I-SPY2^[33]^	Nanda	2020	UK	Doxorubicin + cyclophosphamide + paclitaxel + pembrolizumab; doxorubicin + cyclophosphamide + paclitaxel	29; 85	Pembrolizumab: 200 mg iv cycles every 3 weeks, paclitaxel: 80 mg/m^2^ iv cycles weekly, doxorubicin: 60 mg/m^2^ iv every 2 or 3 weeks for 4 cycles; cyclophosphamide: 600 mg/m^2^ iv every 2 or 3 weeks for 4 cycles	pCR rate; EFS curve
ABCSG-24^[34]^	Steger	2014	Austria	Epirubicin + docetaxel + capecitabine; epirubicin + docetaxel	64; 63	Six 3-weekly cycles of epirubicin + docetaxel [both 75 mg/m^2^], capecitabine [1000 mg/m^2^, twice daily, days 1–14]	pCR rate
KEYNOTE-522^[35–37]^	Schmid	2020; 2022; 2024	UK	Doxorubicin or epirubicin + cyclophosphamide + paclitaxel + carboplatin + pembrolizumab; doxorubicin or epirubicin + cyclophosphamide + paclitaxel + carboplatin	784; 390	Pembrolizumab [200 mg] every 3 weeks, paclitaxel [80 mg/m^2^ weekly], carboplatin [AUC5 every 3 weeks], doxorubicin [60 mg/m^2^] or epirubicin [90 mg/m^2^], cyclophosphamide [600 mg/m^2^ every 3 weeks]	pCR rate; EFS HR and curve; OS HR and curve
GeparSixtoGBG-66^[38,39]^	von Minckwitz; Hahnen	2014; 2017	Germany	Paclitaxel + nonpegylated-liposomal-doxorubicin + carboplatin + bevacizumab; Paclitaxel + nonpegylated-liposomal-doxorubicin + carboplatin	146; 145	Paclitaxel 80 mg/m^2^, nonpegylated liposomal doxorubicin 20 mg/m^2^, both given once a week for 18 weeks, carboplatin received the drug at a dose of AUC2, once every week for 18 weeks, bevacizumab 15 mg/kg iv every 3 weeks simultaneously with all cycles	pCR rate; EFS (DFS) HR and curve
CALGB 40603^[40,41]^	Sikov; Shepherd	2014; 2021	USA	Doxorubicin + cyclophosphamide + paclitaxel; Doxorubicin + cyclophosphamide + paclitaxel + bevacizumab; Doxorubicin + cyclophosphamide + paclitaxel + carboplatin; Doxorubicin + cyclophosphamide + paclitaxel + carboplatin + bevacizumab	107; 105; 111; 110	Paclitaxel 80 mg/m^2^ once per week for 12 weeks, doxorubicin plus cyclophosphamide once every 2 weeks for 4 cycles, carboplatin AUC6 once every 3 weeks for 4 cycles, bevacizumab 10 mg/kg once every 2 weeks for 9 cycles	pCR rate; EFS HR; OS HR
GBG-44^[42]^	Gerber	2013	Germany	Epirubicin + cyclophosphamide + docetaxel + bevacizumab; epirubicin + cyclophosphamide + docetaxel	237; 229	Epirubicin [90 mg/m^2^] plus cyclophosphamide [600 mg/m^2^], both administered on day 1, every 3 weeks for 4 cycles, docetaxel [100 mg/m^2^] on day 1, every 3 weeks, bevacizumab [15 mg/kg] iv every 3 weeks	pCR rate
NeoCART^[10]^	Zhang	2021	China	Docetaxel + carboplatin; epirubicin + cyclophosphamide + docetaxel	44; 44	Docetaxel [75 mg/m^2^] intravenously every 3 weeks, carboplatin AUC6, iv every 3 weeks for 6 cycles, epirubicin [90 mg/m^2^], cyclophosphamide [600 mg/m^2^], every 3 weeks for 4 cycles	pCR rate; EFS HR and curve; OS HR and curve
NeoTRIP^[43]^	Gianni	2022	Italy	Epirubicin + cyclophosphamide + nab-paclitaxel + carboplatin + atezolizumab; epirubicin + cyclophosphamide + nab-paclitaxel + carboplatin	138; 142	Carboplatin AUC2 and nab-paclitaxel 125 mg/m^2^ iv on days 1 and 8, atezolizumab 1200 mg iv on day 1, epirubicin [90 mg/m^2^], cyclophosphamide [600 mg/m^2^], every 3 weeks for 4 cycles	pCR rate
NeoSTOP^[12]^	Sharma	2021	USA	Doxorubicin + cyclophosphamide + paclitaxel + carboplatin; paclitaxel + carboplatin	48; 52	Carboplatin AUC6 every 3 weeks for 4 cycles, paclitaxel 80 mg/m^2^ weekly for 12 weeks, doxorubicin 60 mg/m^2^, cyclophosphamide 600 mg/m^2^ every 2 weeks for 4 cycles	pCR rate; EFS curve; OS curve
SOLTI NeoPARP^[44]^	Llombart-Cussac	2015	Spain	Paclitaxel; paclitaxel + iniparib	47; 94	Paclitaxel 80 mg/m^2^, day 1; or in combination with iniparib, on either a once-weekly 11.2 mg/kg, day 1 or twice-weekly [5.6 mg/kg, days 1 and 4]	pCR rate
NATT^[45]^	Chen	2013	China	Epirubicin or doxorubicin + docetaxel + cyclophosphamide; docetaxel + cyclophosphamide	26; 23	Docetaxel 75 mg/m^2^, epirubicin 60 mg/m^2^ or doxorubicin 50 mg/m^2^, and cyclophosphamide 500 mg/m^2^ on day 1 every 21 days	pCR rate; EFS curve; OS curve
GeparOcto— GBG 84^[46,47]^	Schneeweiss	2019; 2020	Germany	Epirubicin + cyclophosphamide + paclitaxel + carboplatin; epirubicin + cyclophosphamide + paclitaxel	203; 200	Epirubicin 150 mg/m^2^ q2w for 3 cycles, paclitaxel 225 mg/m^2^ q2w for 3 cycles followed by cyclophosphamide 2000 mg/m^2^ q2w for 3 cycles, carboplatin AUC1.5 weekly for 18 weeks	pCR rate; EFS (DFS) HR and curve; OS HR and curve
Daniel^[48]^	Daniel Enriquez	2017	Peru	Carboplatin + docetaxel; adriamycin + cyclophosphamide + paclitaxel	27; 34	Carboplatin AUC6 + docetaxel 75 mg/m^2^ q21d for 6 cycles, adriamycin 60mg/m^2^ + cyclophosphamide 600mg/m^2^ q21d for 4 cycles, weekly paclitaxel 80 mg/m^2^ for 12 weeks	pCR rate
CamRelief^[49]^	Chen	2024	China	Epirubicin + cyclophosphamide + nab-paclitaxel + carboplatin + camrelizumab; epirubicin + cyclophosphamide + nab-paclitaxel + carboplatin	222; 219	Camrelizumab 200 mg, nab-paclitaxel [100 mg/m^2^], carboplatin [AUC1.5] on days 1, 8, and 15 in 28-day cycles for 16 weeks, epirubicin [90 mg/m^2^] and cyclophosphamide [500 mg/m^2^] every 2 weeks for 8 weeks	pCR rate; EFS HR and curve
GBG 44–GeparQuinto^[50]^	von Minckwitz	2014	Germany	Epirubicin + cyclophosphamide + docetaxel + bevacizumab; epirubicin + cyclophosphamide + docetaxel	340; 323	Epirubicin 90 mg/m^2^, cyclophosphamide 600 mg/m^2^, day 1, every 3 weeks, docetaxel 100 mg/m^2^, day 1, every 3 weeks, bevacizumab 15 mg/kg iv every 3 weeks	pCR rate; EFS (DFS) HR
Masashi^[51,52]^	Ando; Iwase	2014; 2020	Japan	Epirubicin + fluorouracil + cyclophosphamide + paclitaxel + carboplatin; Epirubicin + fluorouracil + cyclophosphamide + paclitaxel	37; 38	Paclitaxel 80 mg/m^2^ days 1, 8, and 15 every 3 weeks for 4 cycles. Carboplatin AUC5 day 1, cyclophosphamide 500 mg/m^2^ + epirubicin 100 mg/m^2^ + fluorouracil 500 mg/m^2^ iv on day 1 every 3 weeks for 4 cycles.	pCR rate; EFS HR and curve; OS HR and curve
NCT00499603^[53]^	Gonzalez-Angulo	2014	USA	Epirubicin + fluorouracil + cyclophosphamide + paclitaxel + everolimus; epirubicin + fluorouracil + cyclophosphamide + paclitaxel	23; 27	Paclitaxel 80 mg/m^2^ weekly for 12 weeks, fluorouracil 500 mg/m^2^, epirubicin 100 mg/m^2^, and cyclophosphamide 500 mg/m^2^ every 3 weeks for 4 cycles; everolimus 30 mg PO weekly for 12 weeks	pCR rate

AUC = area under the curve, DFS = disease free survival, EFS = event free survival, HR = hazards ratio, OS = overall survival, pCR = pathological complete response, RFS = relapse free survival.

### 3.2. Primary endpoint

**Figure 2.**
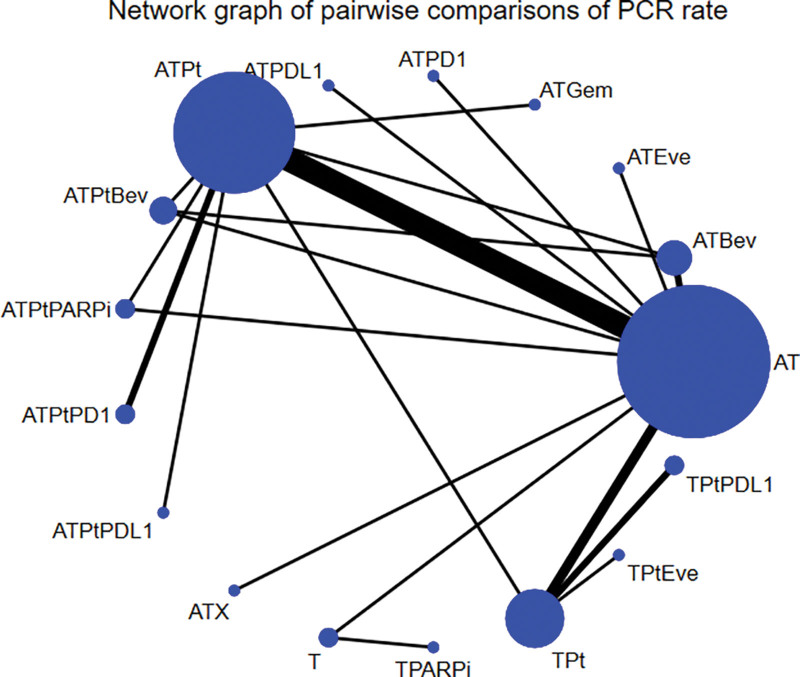
Network diagram of eligible comparisons included in the network meta-analysis for pCR rate. AT = containing anthracyclines and taxanes, ATBev = containing anthracyclines, taxanes, and bevacizumab, ATEve = containing anthracyclines, taxanes, and everolimus, ATGem = containing anthracyclines, taxanes, and gemcitabine, ATPD1 = containing anthracyclines, taxanes, and PD-1, ATPDL1 = containing anthracyclines, taxanes, and PD-L1, ATPt = containing anthracyclines, taxanes, and platinum, ATPtBev = containing anthracyclines, taxanes, platinum, and bevacizumab, ATPtPARPi = containing anthracyclines, taxanes, platinum, and PARPi, ATPtPD1 = containing anthracyclines, taxanes, platinum, and PD-1, ATPtPDL1 = containing anthracyclines, taxanes, platinum, and PD-L1, ATX = containing anthracyclines, taxanes, and capacitabine, pCR = pathological complete response, T, containing taxanes only, TPARPi, containing taxanes and PARPi, TPt = containing taxanes and platinum, TPtEve = containing taxanes, platinum, and everolimus, TPtPDL1 = containing taxanes, platinum, and PD-L1.

Figure 2 shows the network graphs of pairwise comparison of regimens on pCR rate. Compared with AT, only ATPtPD1 (OR = 5.68, 95% CI: 1.49–21.62) significantly increased the pCR rate (Table S1, Supplemental Digital Content, https://links.lww.com/MD/R101)

### 3.3. Second endpoint

#### 3.3.1. EFS at each time point

**Figure 3.**
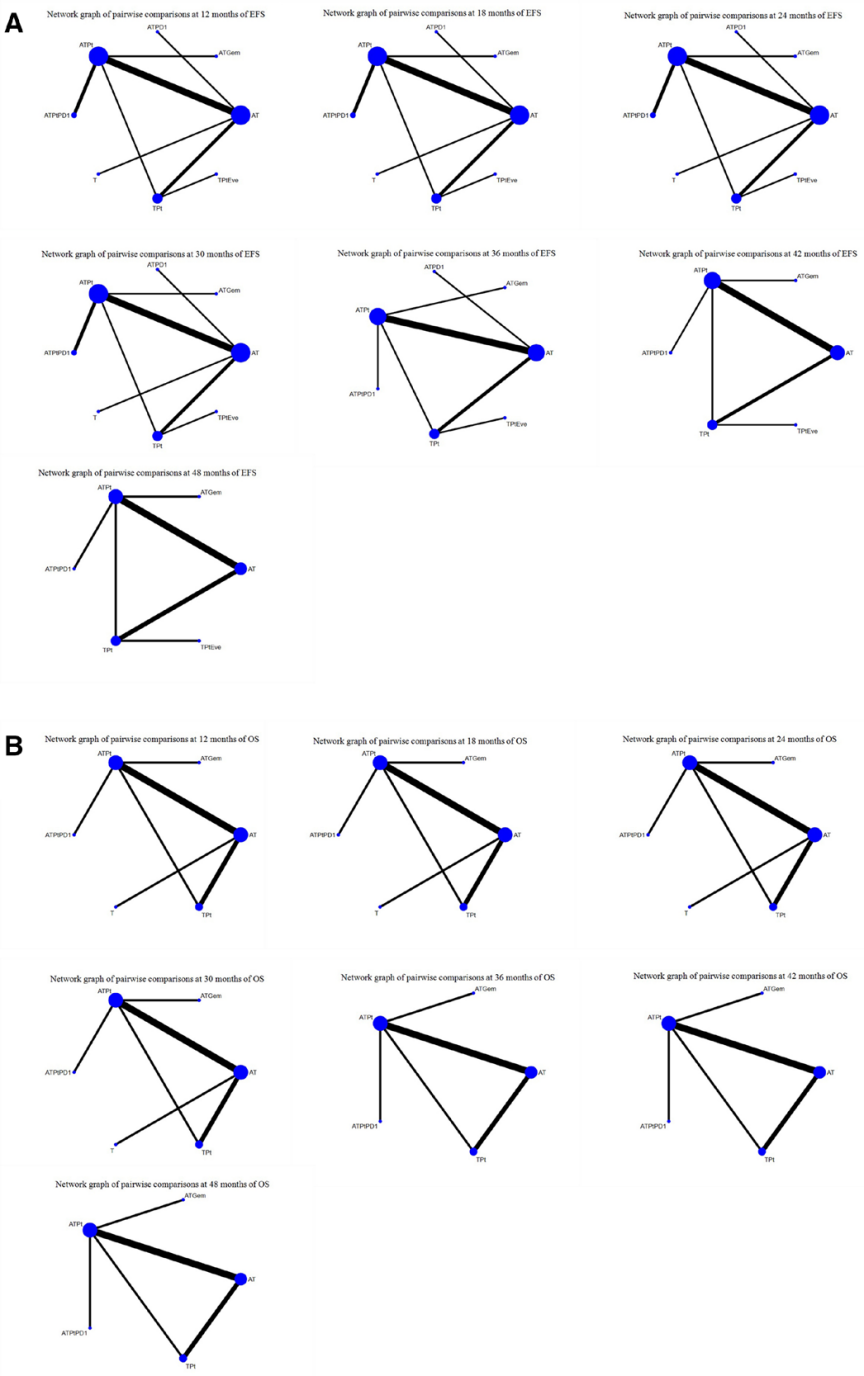
Network graphs of the pairwise comparisons of regimens at each time point of the (A) EFS curve and (B) OS curve. AT = containing anthracyclines and taxanes, ATGem = containing anthracyclines, taxanes, and gemcitabine, ATPD1 = containing anthracyclines, taxanes, and PD-1, ATPt = containing anthracyclines, taxanes, and platinum, ATPtPD1 = containing anthracyclines, taxanes, platinum, and PD-1, EFS = events free survival, OS = overall survival, T = containing taxanes only, TPt = containing taxanes and platinum, TPtEve = containing taxanes, platinum, and everolimus.

Figure 3A shows the network graphs of pairwise comparison of regimens on each time point of the EFS.

At 12th month, compared with AT, there was no treatment significantly increased the 12 months EFS rate (Table S2A, Supplemental Digital Content, https://links.lww.com/MD/R101).

At 18th month, compared with AT, only ATPtPD1 (OR = 2.28, 95% CI: 1.24–4.19), significantly increased the 18 months EFS rate (Table S2B, Supplemental Digital Content, https://links.lww.com/MD/R101).

At 24th month, compared with AT, only ATPtPD1 (OR = 2.43, 95% CI: 1.01–5.89), significantly increased the 24 months EFS rate (Table S2C, Supplemental Digital Content, https://links.lww.com/MD/R101).

At 30th month, compared with AT, only ATPtPD1 (OR = 3.021, 95% CI: 1.40–7.38), significantly increased the 30 months EFS rate (Table S2D, Supplemental Digital Content, https://links.lww.com/MD/R101).

At 36th month, compared with AT, ATPtPD1 (OR = 4.23, 95% CI: 1.15–15.57), and ATPt (OR = 2.54, 95% CI: 1.16–5.57). ATPtPD1, as the top SUCRA-ranked intervention, did not show significant advantage when compared with ATPt (Table S2E, Supplemental Digital Content, https://links.lww.com/MD/R101).

At 42nd month, compared with AT, ATPtPD1 (OR = 4.62, 95% CI: 1.38–15.50), and ATPt (OR = 2.68, 95% CI: 1.27–5.69). ATPtPD1, as the top SUCRA-ranked intervention, did not show significant advantage when compared with ATPt (Table S2F, Supplemental Digital Content, https://links.lww.com/MD/R101).

At 48th month, compared with AT, ATPtPD1 (OR = 4.04, 95% CI: 1.47–11.13), and ATPt (OR = 2.18, 95% CI: 1.12–4.23). ATPtPD1, as the top SUCRA-ranked intervention, did not show significant advantage when compared with ATPt (Table S2G, Supplemental Digital Content, https://links.lww.com/MD/R101).

As described, the regimen with the best significant effect on EFS from 12 to 48 months compared to AT was ATPtPD1 (Table 2).

**Table 2:** Results for each methods that were significant compared with AT (shown as odds/hazard ratio and 95% confidence intervals).

Methods	Control group	ATPtPD1	ATPt	ATPtPARPi
pCR rate	AT	5.68 (1.49–21.62)	X	X
12M EFS	AT	X	X	NR
18M EFS	AT	2.28 (1.24–4.19)	X	NR
24M EFS	AT	2.43 (1.01–5.89)	X	NR
30M EFS	AT	3.21 (1.40–7.38)	X	NR
36M EFS	AT	4.23 (1.15–15.57)	2.54 (1.16–5.57)	NR
42M EFS	AT	4.62 (1.38–15.50)	2.68 (1.27–5.69)	NR
48M EFS	AT	4.04 (1.47–11.13)	2.18 (1.12–4.23)	NR
12M OS	AT	X	X	NR
18M OS	AT	3.56 (1.33–9.52)	2.72 (1.18–6.28)	NR
24M OS	AT	X	X	NR
30M OS	AT	2.49 (1.27–4.87)	1.98 (1.15–3.40)	NR
36M OS	AT	2.49 (1.34–4.65)	1.94 (1.18–3.19)	NR
42M OS	AT	3.17 (1.74–5.78)	2.32 (1.43–3.77)	NR
48M OS	AT	2.97 (1.12–7.91)	X	NR
HR for EFS	AT	2.24 (1.42–3.59)	1.53 (1.17–2.08)	1.64 (1.1–2.53)
Adjusted HR for EFS	AT	2.29 (1.4–3.87)	1.57 (1.17–2.22)	1.66 (1.08–2.65)
HR for OS	AT	2.67 (1.03–7.35)	X	X
Adjusted HR for OS	AT	2.71 (1.19–6.39)	X	X

AT = containing anthracyclines and taxanes, ATPt = containing anthracyclines, taxanes, and platinum, ATPtPARPi = containing anthracyclines, taxanes, platinum, and PARPi, ATPtPD1 = containing anthracyclines, taxanes, platinum, and PD-1, EFS = events free survival, HR = hazard ratio, M = month, NR = not reported, OS = overall survival, pCR = pathological complete response, X = significant advantage.

#### 3.3.2. OS at each time point

Figure 3B shows the network graphs of pairwise comparison of regimens on each time point of the OS.

At 12th month, compared with AT, there was no treatment significantly increased the 12 months OS rate (Table S3A, Supplemental Digital Content, https://links.lww.com/MD/R101).

At 18th month, compared with AT, ATPtPD1 (OR = 3.56, 95% CI: 1.33–9.52), and ATPt (OR = 2.72, 95% CI: 1.18–6.28). ATPtPD1, as the top SUCRA-ranked intervention, did not show significant advantage when compared with ATPt (Table S3B, Supplemental Digital Content, https://links.lww.com/MD/R101).

At 24th month, compared with AT, there was no treatment significantly increased the 24 months OS rate (Table S3C, Supplemental Digital Content, https://links.lww.com/MD/R101).

At 30th month, compared with AT, ATPtPD1 (OR = 2.49, 95% CI: 1.27–4.78), and ATPt (OR = 1.98, 95% CI: 1.15–3.40). ATPtPD1, as the top SUCRA-ranked intervention, did not show significant advantage when compared with ATPt (Table S3D, Supplemental Digital Content, https://links.lww.com/MD/R101).

At 36th month, compared with AT, ATPtPD1 (OR = 2.49, 95% CI: 1.34–4.65), and ATPt (OR = 1.94, 95% CI: 1.18–3.19). ATPtPD1, as the top SUCRA-ranked intervention, did not show significant advantage when compared with ATPt (Table S3E, Supplemental Digital Content, https://links.lww.com/MD/R101).

At 42nd month, compared with AT, ATPtPD1 (OR = 3.17, 95% CI: 1.74–5.78), and ATPt (OR = 2.32, 95% CI: 1.43–3.77). ATPtPD1, as the top SUCRA-ranked intervention, did not show significant advantage when compared with ATPt (Table S3F, Supplemental Digital Content, https://links.lww.com/MD/R101).

At 48th month, compared with AT, only ATPtPD1 (OR = 2.97, 95% CI: 1.12–7.91), showed the significant advantage on the 48 months OS rate (Table S3G, Supplemental Digital Content, https://links.lww.com/MD/R101).

As described, the regimen with the best significant effect on OS from 12 to 48 months compared to AT was ATPtPD1 (Table 2).

### 3.4. Tertiary endpoints

#### 3.4.1. HR for EFS and regression analysis

**Figure 4.**
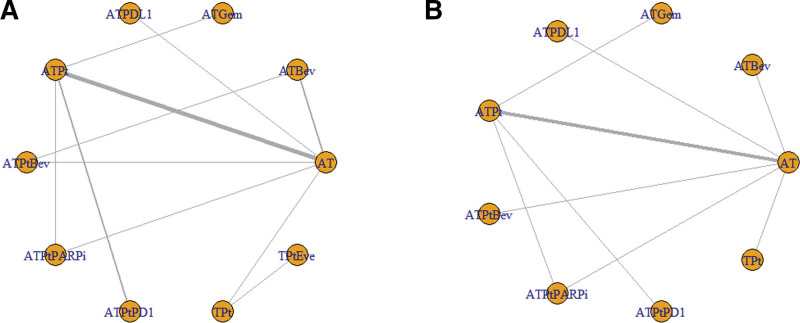
Network meta‑analysis plots for HR of (A) events free survival and (B) overall survival. AT = containing anthracyclines and taxanes, ATBev = containing anthracyclines, taxanes, and bevacizumab, ATGem = containing anthracyclines, taxanes, and gemcitabine, ATPDL1 = containing anthracyclines, taxanes, and PD-L1, ATPt = containing anthracyclines, taxanes, and platinum, ATPtBev = containing anthracyclines, taxanes, platinum, and bevacizumab, ATPtPARPi = containing anthracyclines, taxanes, platinum, and PARPi, ATPtPD1 = containing anthracyclines, taxanes, platinum, and PD-1, HR = hazard ratio, TPt = containing taxanes and platinum, TPtEve = containing taxanes, platinum, and everolimus.

Due to limited EFS data, we treated data regarding disease-free survival and relapse-free survival reported in these studies as EFS data. A total of 13/25 trails reported outcomes associated with the HRs of EFS. The 10 included interventions were compared directly and indirectly (Fig. 4A). All articles reported the median follow-up time.

In the raw analysis: compared with AT, ATPtPD1 (HR = 2.24, 95% CI: 1.42–3.59), ATPtPARPi (HR = 1.64, 95% CI: 1.10–2.53), and ATPt (HR = 1.53, 95% CI: 1.17–2.08) showed significant advantage, as shown in Table S4A, Supplemental Digital Content, https://links.lww.com/MD/R101.

After the regression analysis: compared with AT, ATPtPD1 (HR = 2.29, 95% CI: 1.40–3.87), ATPtPARPi (HR = 1.66, 95% CI: 1.08–2.65), and ATPt (HR = 1.57, 95% CI: 1.17–2.22) showed significant advantage, as shown in Table S4B, Supplemental Digital Content, https://links.lww.com/MD/R101.

**Figure 5.**
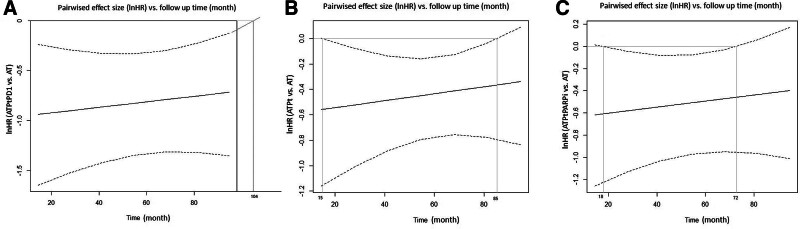
Curves depicting pairwise comparisons of effect sizes (lnHR) versus follow-up time for events free survival. (A) ATPtPD1, containing anthracyclines, taxanes, platinum, and PD-1; (B) ATPt, containing anthracyclines, taxanes, and platinum; (C) ATPtPARPi, containing anthracyclines, taxanes, platinum, and PARPi. AT = containing anthracyclines and taxanes.

Time window analysis: when compared with AT, ATPtPD1 showed significant advantage in the time window of 0 to 104 months after the initiation of treatment (Fig. 5A). The time window for ATPt was 15 to 85 months(Fig. 5B) and for ATPtPARPi was 18 to 72 months (Fig. 5C).

As described, the regimen with the best significant effect on HR for EFS compared to AT was ATPtPD1 (Table 2).

#### 3.4.2. HR for OS and regression analysis

A total of 9/25 trails reported outcomes associated with the HRs of OS. The 9 included interventions were compared directly and indirectly (Fig. 4B). All articles reported the median follow-up time.

In the raw analysis: compared with AT, only ATPtPD1 (HR = 2.67, 95% CI: 1.03–7.35) showed significant advantage, as shown in Table S5A, Supplemental Digital Content, https://links.lww.com/MD/R101.

After the regression analysis: compared with AT, only ATPtPD1 (HR = 2.71, 95% CI: 1.19–6.39) showed significant advantage, as shown in Table S5B, Supplemental Digital Content, https://links.lww.com/MD/R101.

**Figure 6.**
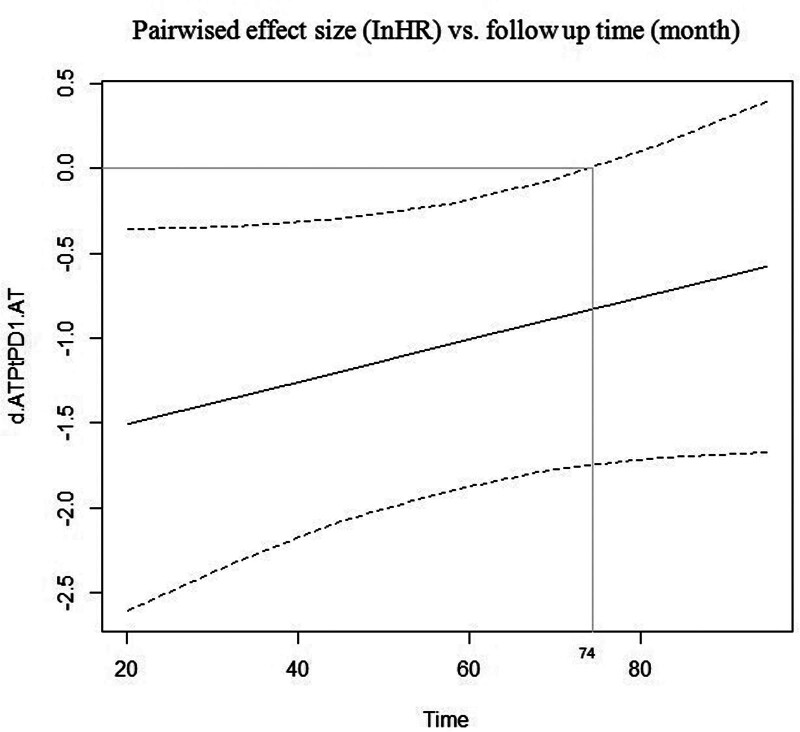
Curves depicting pairwise comparisons of effect sizes (lnHR) versus follow-up time for overall survival. AT = containing anthracyclines and taxanes, ATPtPD1 = containing anthracyclines, taxanes, platinum, and PD-1.

Time window analysis: when compared with AT, ATPtPD1 showed significant advantage in the time window of 0 to 74 months after the initiation of treatment (Fig. 6).

As described, the regimen with the best significant effect on HR for OS compared to AT was ATPtPD1 (Table 2).

### 3.5. Inconsistency tests and heterogeneity analysis and small sample effect tests

There was no heterogeneity between studies included in the present Bayesian NMA and network regression analysis (Figures S2–S9, Supplemental Digital Content, https://links.lww.com/MD/R101). Small sample effect was explored by network funnel plot. *P* < .05 was considered to be statistically significant (Figures S10–S12, Supplemental Digital Content, https://links.lww.com/MD/R101).

## 4. Discussion

In recent years, various advanced diagnostic technologies have emerged, making more and more breast cancer found at early stagey.^[54,55]^ Early breast cancer can improve the OS of the overall diseased population, especially in the hormone receptor positive and human epidermal growth factor receptor 2 positive subgroups. Currently, TNBC lacks targeted therapeutic targets, resulting in limited overall efficacy. TNBC is an aggressive form of breast cancer that is often associated with poor patient outcomes, largely due to the limited treatment options available.^[56,57]^ Most TNBC patients with early-stage are now treated with neoadjuvant chemotherapy. This approach, primarily driven by the significant prognostic benefit associated with pCR, particularly in patients with more aggressive subtypes, has become the standard of treatment. New treatment methods, such as immunotherapy, have achieved definite results in the application of TNBC, improving EFS and OS.^[58]^ Therefore, in the treatment of early TNBC, immunotherapy has been seeking a breakthrough. Some of these new treatments have significantly improved pCR, but have not effectively improved EFS or OS.^[59]^ Such treatments do not fully comply with the principles and objectives of tumor treatment, so we do not recommend them. The aim of the present study was to discuss the best options for neoadjuvant chemotherapy of TNBC.

To the best of our knowledge, the present study reports the first Bayesian NMA and regression analysis comparing relative efficacy of all current available neoadjuvant therapies for TNBC. The findings were as follows.

### 4.1. Core findings

For pCR, only ATPtPD1 can improve results compared with AT.

For EFS with a follow-up period of 12th to 48th month, ATPtPD1 demonstrated a significant advantage from 18th to 48th month compared with AT. The significant result of ATPt also showed, but only between 36th and 48th month after initial treatment.

For OS with a follow-up period of 12th to 48th month, when compared with AT, ATPtPD1 and ATPt both showed a significant efficacy only on the 18th month, while there is no improvement on the 12th and 24th months. However, at subsequent follow-up times, significant efficacy was found for ATPtPD1 extending from 30th to 48th months, whereas ATPt only extended from 30th to 42nd months.

For HR of EFS: ATPtPD1, ATPt, and ATPtPARPi demonstrated a significant advantage compared with AT. After regression analysis using follow-up time as a correcting factor, the significant improvement did not change significantly. However, from the time window analysis, ATPtPD1 showed significant advantage in a longer time window of 0 to 104 months after the initiation of treatment. While the time window for ATPt was 15 to 85 months, for ATPtPARPi it was 18 to 72 months.

For HR for OS: only ATPtPD1 significantly improves HR for OS compared with AT. Similarly, after regression analysis using follow-up time as a correcting factor, the significant improvement does not change significantly. ATPtPD1 was the only regimen that shows significant efficacy in pCR rate, EFS/OS during the follow-up period of 18 to 48 months after treatment, and HR for EFS/OS.

### 4.2. Clinical-pathological correlation

Anthracyclines insert themselves between DNA base pairs (intercalation), disrupting the DNA helix. This interference inhibits essential processes like DNA replication and RNA transcription, critical for rapidly dividing cancer cells. Anthracyclines undergo redox cycling, producing reactive oxygen species (ROS) such as superoxide radicals. These ROS induce oxidative stress, damaging DNA, proteins, and lipids. Cancer cells, often with weaker antioxidant defenses, are particularly vulnerable.^[60]^ Iron-anthracycline complexes can exacerbate ROS production, contributing to both efficacy and side effects like cardiotoxicity.^[60]^ Recent research explores strategies to mitigate toxicity while enhancing efficacy, such as liposomal formulations or targeting tumor-specific pathways.^[61]^

The antitumor mechanisms of taxanes (e.g., paclitaxel, docetaxel, cabazitaxel) primarily involve disrupting microtubule dynamics, which are critical for cell division and survival. Taxanes bind to β-tubulin subunits of microtubules, stabilizing them and preventing their disassembly (depolymerization). This disrupts the normal dynamic instability of microtubules, a process required for proper mitotic spindle formation during cell division. Cells become trapped in metaphase arrest (mitotic arrest), unable to complete chromosome segregation. Prolonged mitotic arrest triggers apoptosis (programmed cell death).^[62]^ Taxanes also affect interphase microtubules, interfering with critical cellular functions in nondividing cells, such as intracellular transport (e.g., organelle movement, vesicle trafficking). Cells signaling pathways dependent on microtubule integrity. This broad disruption contributes to tumor cell death even in slowly proliferating cancers.^[63]^

The antitumor mechanisms of platinum-based chemotherapy drugs (e.g., cisplatin, carboplatin, oxaliplatin, lobaplatin) primarily involve DNA damage and disruption of cellular processes, leading to apoptosis (programmed cell death). The activated platinum binds to purine bases (mainly guanine) in DNA, including intrastrand crosslink and interstrand crosslink.^[64]^ Finally, damaged DNA stalls replication forks, triggering cell cycle arrest (G2/M phase). Platinum-induced tumor cell death can release damage-associated molecular patterns, activating dendritic cells and enhancing antitumor immunity.^[64,65]^

The antitumor mechanism of PD-1/PD-L1 inhibitors (e.g., pembrolizumab, camrelizumab, atelizumab) involves disrupting immune evasion strategies employed by cancer cells, thereby reactivating T-cell-mediated tumor destruction.^[66]^ PD-1 is a checkpoint receptor on activated T-cells that suppresses their activity when engaged. PD-L1 is a ligand often overexpressed by tumor cells and stromal cells in the tumor microenvironment, which binds to PD-1 to inhibit T-cell function.^[67]^ Deregulation of T-cell inhibition as a core antitumor mechanism in immunotherapy. Tumors exploit the PD-1/PD-L1 pathway by upregulating PD-L1 expression. When PD-L1 binds to PD-1 on tumor-infiltrating T-cells, it delivers a co-inhibitory signal. After reducing proliferation, cytokine production, and cytotoxic activity, T-cell function is restored and kills tumor cells directly.^[67]^

PD-1 acts as a receptor and receives inhibitory signals directly from PD-L1/PD-L2. PD-L1 acts as a ligand and transmits inhibitory signals by binding to PD-1. Tumor cells directly inhibit T cell function through high expression of PD-L1, while inducing immunosuppressive activity of regulatory T cells. PD-1 inhibitors (e.g., pembrolizumab, camrelizumab) directly block the PD-1 receptor, preventing it from binding to PD-L1/PD-L2, and may also affect the 2 inhibitory pathways, PD-L1 and PD-L2. PD-L1 inhibitors (e.g., atelizumab) block PD-L1 only, preserving PD-L2 interaction with PD-1 (may reduce some autoimmune side effects). This is more specific for tumors with high PD-L1 expression.^[68]^ PD-1 inhibitors may block the dual binding of PD-1 to PD-L1/PD-L2, leading to more extensive immune activation and a slightly higher incidence of side effects such as pneumonia and thyroiditis. PD-L1 inhibitors block only PD-L1, preserving PD-L2 binding to PD-1 (PD-L2 is expressed in some normal tissues), which may theoretically reduce some side effects, but the clinical differences are not yet significant.^[69]^

### 4.3. Feasibility analysis

Chemotherapy containing anthracycline, taxanes, and platinum has been applied to breast cancer for a long time. The most common toxic side effects are bone marrow suppression, nausea and vomiting, and fatigue.^[70]^ Colonizing human granulocyte stimulating factor is effective in ameliorating neutropenia induced during chemotherapy.^[71]^ And nausea and vomiting are well controlled after application of a standard antiemetic protocol.^[72]^ According to relevant trails, treatments combined with PD1 inhibitors did not increase in chemotherapy related toxic side effects. Only exhibiting adverse reactions related to immunotherapy like rash, interstitial pneumonia, and thyroid dysfunction. But all of adverse reactions related to immunotherapy were controllable and relievable.^[73]^ To avoid excessive accumulation of toxic side effects, sequential administration can be used in order to be better accepted among patients.^[74]^

### 4.4. Limitations

First, the sample sizes of certain included studies were inadequate, resulting in the small sample effect and potential bias. Second, in the analyses of HR EFS/OS, certain studies had not reported 95% CIs or the interquartile range. These studies had to be excluded, which potentially shrank the sample size by another means, eventually increasing random error. Third, the limited resolution of survival curve images in certain studies was compromised. Finally, the quality of some of the studies was low, bringing potential interference.

### 4.5. Perspective

We hope that the design of future clinical trials will be more precise and the final OS data will be reported. The data from subgroup analysis should be more detailed. Adverse events should be evaluated after long-term follow-ups and could be compared at each time node. When making clinical decisions, adverse effects should be considered. FDA Adverse Event Reporting System database can provide some information on these adverse events.

## 5. Conclusion

In conclusion, considering the benefit of treatment on pCR rate and EFS/OS emerged earlier and over a long period of time, ATPtPD1 should be recommended as the optimal neoadjuvant therapy in TNBC. However, it is necessary to design more RCTs to confirm this result.

## Author contributions

**Conceptualization:** Yicheng Jiang.

**Data curation:** Wenbo Deng, Meng Xu, Qiang Wang.

**Formal analysis:** Yicheng Jiang, Jiajia Zeng, Meng Xu.

**Methodology:** Jian Liu, Ruijun Tang.

**Software:** Yicheng Jiang, Jiajia Zeng, Ruijun Tang, Qiang Wang.

**Writing – original draft:** Wenjie Shi.

**Writing – review & editing:** Jian Liu, Ulf Dietrich Kahlert, Wenjie Shi, Qiang Wang.

## Supplementary Material



## References

[1] Giaquinto AN Sung H Newman LA. Breast cancer statistics 2024. CA Cancer J Clin. 2024;74:477–95. 39352042 10.3322/caac.21863

[2] Kumar P Aggarwal R. An overview of triple-negative breast cancer. Arch Gynecol Obstet. 2016;293:247–69. 26341644 10.1007/s00404-015-3859-y

[3] Berry DA Cirrincione C Henderson IC. Estrogen-receptor status and outcomes of modern chemotherapy for patients with node-positive breast cancer. JAMA. 2006;295:1658–67. 16609087 10.1001/jama.295.14.1658 PMC1459540

[4] Carey LA Dees EC Sawyer L. The triple negative paradox: primary tumor chemosensitivity of breast cancer subtypes. Clin Cancer Res. 2007;13:2329–34. 17438091 10.1158/1078-0432.CCR-06-1109

[5] Asselain B Barlow W Bartlett J. Long-term outcomes for neoadjuvant versus adjuvant chemotherapy in early breast cancer: meta-analysis of individual patient data from ten randomised trials. Lancet Oncol. 2018;19:27–39. 29242041 10.1016/S1470-2045(17)30777-5 PMC5757427

[6] Gradishar WJ Moran MS Abraham J. Breast cancer, version 3.2024, NCCN clinical practice guidelines in oncology. J Natl Compr Canc Netw. 2024;22:331–57. 39019058 10.6004/jnccn.2024.0035

[7] Loibl S André F Bachelot T. Early breast cancer: ESMO clinical practice guideline for diagnosis, treatment and follow-up. Ann Oncol. 2024;35:159–82. 38101773 10.1016/j.annonc.2023.11.016

[8] Burstein H Curigliano G Thürlimann B. Customizing local and systemic therapies for women with early breast cancer: the St. Gallen International Consensus Guidelines for treatment of early breast cancer 2021. Ann Oncol. 2021;32:1216–35. 34242744 10.1016/j.annonc.2021.06.023 PMC9906308

[9] Yan W Wu X Wang S. Lobaplatin-based neoadjuvant chemotherapy for triple-negative breast cancer: a 5-year follow-up of a randomized, open-label, phase II trial. Ther Adv Med Oncol. 2022;14:1–10. 10.1177/17588359221107111 PMC9234826 35769355

[10] Zhang L Wu ZY Li J. Neoadjuvant docetaxel plus carboplatin vs epirubicin plus cyclophosphamide followed by docetaxel in triple‐negative, early‐stage breast cancer (NeoCART): results from a multicenter, randomized controlled, open‐label phase II trial. Int J Cancer. 2021;150:654–62. 34591977 10.1002/ijc.33830

[11] Ahmad S. Platinum–DNA interactions and subsequent cellular processes controlling sensitivity to anticancer platinum complexes. Chem Biodivers. 2010;7:543–66. 20232326 10.1002/cbdv.200800340

[12] Sharma P Kimler BF O’Dea A. Randomized phase II trial of anthracycline-free and anthracycline-containing neoadjuvant carboplatin chemotherapy regimens in stage I–III triple-negative breast cancer (NeoSTOP). Clin Cancer Res. 2021;27:975–82. 33208340 10.1158/1078-0432.CCR-20-3646 PMC7887017

[13] Brown JS Kaye SB Yap TA. PARP inhibitors: the race is on. Br J Cancer. 2016;114:713–5. 27022824 10.1038/bjc.2016.67 PMC4984871

[14] LeVasseur N Sun J Gondara L. Impact of pathologic complete response on survival after neoadjuvant chemotherapy in early-stage breast cancer: a population-based analysis. J Cancer Res Clin Oncol. 2020;146:529–36. 31741041 10.1007/s00432-019-03083-y PMC11804444

[15] Bear HD Tang G Rastogi P. Bevacizumab added to neoadjuvant chemotherapy for breast cancer. N Engl J Med. 2012;366:310–20. 22276821 10.1056/NEJMoa1111097 PMC3401076

[16] Bates DO. Vascular endothelial growth factors and vascular permeability. Cardiovasc Res. 2010;87:262–71. 20400620 10.1093/cvr/cvq105 PMC2895541

[17] Nanda R Chow LQ Dees EC. Pembrolizumab in patients with advanced triple-negative breast cancer: phase Ib KEYNOTE-012 study. J Clin Oncol. 2016;34:2460–7. 27138582 10.1200/JCO.2015.64.8931 PMC6816000

[18] Farkona S Diamandis EP Blasutig IM. Cancer immunotherapy: the beginning of the end of cancer? BMC Med. 2016;14:1–18. 27151159 10.1186/s12916-016-0623-5 PMC4858828

[19] Emens LA. Breast cancer immunotherapy: facts and hopes. Clin Cancer Res. 2018;24:511–20. 28801472 10.1158/1078-0432.CCR-16-3001 PMC5796849

[20] Li J Shen G Wang M. Comparative efficacy and safety of first-line neoadjuvant treatments in triple-negative breast cancer: systematic review and network meta-analysis. Clin Exp Med. 2023;23:1489–99. 36152119 10.1007/s10238-022-00894-1

[21] Liu Z Li J Zhao F. Long-term survival after neoadjuvant therapy for triple-negative breast cancer under different treatment regimens: a systematic review and network meta-analysis. BMC Cancer. 2024;24:440–56. 38594636 10.1186/s12885-024-12222-9 PMC11005293

[22] Page MJ McKenzie JE Bossuyt PM. The PRISMA 2020 statement: an updated guideline for reporting systematic reviews. BMJ. 2021;372:n71. 33782057 10.1136/bmj.n71 PMC8005924

[23] Loibl S O’Shaughnessy J Untch M. Addition of the PARP inhibitor veliparib plus carboplatin or carboplatin alone to standard neoadjuvant chemotherapy in triple-negative breast cancer (BrighTNess): a randomised, phase 3 trial. Lancet Oncol. 2018;19:497–509. 29501363 10.1016/S1470-2045(18)30111-6

[24] Loibl S Sikov W Huober J. 119O Event-free survival (EFS), overall survival (OS), and safety of adding veliparib (V) plus carboplatin (Cb) or carboplatin alone to neoadjuvant chemotherapy in triple-negative breast cancer (TNBC) after ≥4 years of follow-up: BrighTNess, a randomized phase III trial. Ann Oncol. 2021;32:S408.

[25] Wu X Tang P Li S. A randomized and open-label phase II trial reports the efficacy of neoadjuvant lobaplatin in breast cancer. Nat Commun. 2018;9:832–40. 29483583 10.1038/s41467-018-03210-2 PMC5827032

[26] Jovanović B Mayer IA Mayer EL. A randomized phase II neoadjuvant study of cisplatin, paclitaxel with or without everolimus in patients with stage II/III Triple-Negative Breast Cancer (TNBC): responses and long-term outcome correlated with increased frequency of DNA damage response gene mutations, TNBC subtype, AR status, and Ki67. Clin Cancer Res. 2017;23:4035–45. 28270498 10.1158/1078-0432.CCR-16-3055 PMC5540799

[27] Alba E Chacon JI Lluch A. A randomized phase II trial of platinum salts in basal-like breast cancer patients in the neoadjuvant setting. Results from the GEICAM/2006-03, multicenter study. Breast Cancer Res Treat. 2012;136:487–93. 23053638 10.1007/s10549-012-2100-y

[28] Mittendorf EA Zhang H Barrios CH. Neoadjuvant atezolizumab in combination with sequential nab-paclitaxel and anthracycline-based chemotherapy versus placebo and chemotherapy in patients with early-stage triple-negative breast cancer (IMpassion031): a randomised, double-blind, phase 3 trial. Lancet. 2020;396:1090–100. 32966830 10.1016/S0140-6736(20)31953-X

[29] Ademuyiwa FO Gao F Street CR. A randomized phase 2 study of neoadjuvant carboplatin and paclitaxel with or without atezolizumab in triple negative breast cancer (TNBC) – NCI 10013. npj Breast Cancer. 2022;8:134. 36585404 10.1038/s41523-022-00500-3 PMC9803651

[30] Zhang P Yin Y Mo H. Better pathologic complete response and relapse-free survival after carboplatin plus paclitaxel compared with epirubicin plus paclitaxel as neoadjuvant chemotherapy for locally advanced triple-negative breast cancer: a randomized phase 2 trial. Oncotarget. 2016;7:60647–56. 27447966 10.18632/oncotarget.10607 PMC5312408

[31] Gluz O Nitz U Liedtke C. Comparison of neoadjuvant nab-paclitaxel+carboplatin vs nab-paclitaxel+gemcitabine in triple-negative breast cancer: randomized WSG-ADAPT-TN trial results. J Natl Cancer Inst. 2018;110:628–37. 29228315 10.1093/jnci/djx258

[32] Gluz O Nitz U Kolberg-Liedtke C. De-escalated neoadjuvant chemotherapy in early Triple-Negative Breast Cancer (TNBC): impact of molecular markers and final survival analysis of the WSG-ADAPT-TN trial. Clin Cancer Res. 2022;28:4995–5003. 35797219 10.1158/1078-0432.CCR-22-0482

[33] Nanda R Liu MC Yau C. Effect of pembrolizumab plus neoadjuvant chemotherapy on pathologic complete response in women with early-stage breast cancer: an analysis of the ongoing phase 2 adaptively randomized I-SPY2 trial. JAMA Oncol. 2020;6:676–84. 32053137 10.1001/jamaoncol.2019.6650 PMC7058271

[34] Steger GG Greil R Lang A. Epirubicin and docetaxel with or without capecitabine as neoadjuvant treatment for early breast cancer: final results of a randomized phase III study (ABCSG-24). Ann Oncol. 2014;25:366–71. 24347519 10.1093/annonc/mdt508

[35] Schmid P Cortes J Pusztai L. Pembrolizumab for early triple-negative breast cancer. N Engl J Med. 2020;382:810–21. 32101663 10.1056/NEJMoa1910549

[36] Schmid P Cortes J Dent R. Event-free survival with pembrolizumab in early triple-negative breast cancer. N Engl J Med. 2022;386:556–67. 35139274 10.1056/NEJMoa2112651

[37] Schmid P Cortes J Dent R. Overall survival with pembrolizumab in early-stage triple-negative breast cancer. N Engl J Med. 2024;391:1981–91. 39282906 10.1056/NEJMoa2409932

[38] von Minckwitz G Schneeweiss A Loibl S. Neoadjuvant carboplatin in patients with triple-negative and HER2-positive early breast cancer (GeparSixto; GBG 66): a randomised phase 2 trial. Lancet Oncol. 2014;15:747–56. 24794243 10.1016/S1470-2045(14)70160-3

[39] Hahnen E Lederer B Hauke J. Germline mutation status, pathological complete response, and disease-free survival in triple-negative breast cancer. JAMA Oncol. 2017;3:1378–85. 28715532 10.1001/jamaoncol.2017.1007 PMC5710508

[40] Sikov WM Berry DA Perou CM. Impact of the addition of carboplatin and/or bevacizumab to neoadjuvant once-per-week paclitaxel followed by dose-dense doxorubicin and cyclophosphamide on pathologic complete response rates in stage II to III triple-negative breast cancer: CALGB 40603 (Alliance). J Clin Oncol. 2015;33:13–21. 25092775 10.1200/JCO.2014.57.0572 PMC4268249

[41] Shepherd JH Ballman K Polley M-YC. CALGB 40603 (Alliance): long-term outcomes and genomic correlates of response and survival after neoadjuvant chemotherapy with or without carboplatin and bevacizumab in triple-negative breast cancer. J Clin Oncol. 2022;40:1323–34. 35044810 10.1200/JCO.21.01506 PMC9015203

[42] Gerber B Loibl S Eidtmann H. Neoadjuvant bevacizumab and anthracycline–taxane-based chemotherapy in 678 triple-negative primary breast cancers; results from the geparquinto study (GBG 44). Ann Oncol. 2013;24:2978–84. 24136883 10.1093/annonc/mdt361

[43] Gianni L Huang CS Egle D. Pathologic complete response (pCR) to neoadjuvant treatment with or without atezolizumab in triple-negative, early high-risk and locally advanced breast cancer: NeoTRIP Michelangelo randomized study. Ann Oncol. 2022;33:534–43. 35182721 10.1016/j.annonc.2022.02.004

[44] Llombart-Cussac A Bermejo B Villanueva C. SOLTI NeoPARP: a phase II randomized study of two schedules of iniparib plus paclitaxel versus paclitaxel alone as neoadjuvant therapy in patients with triple-negative breast cancer. Breast Cancer Res Treat. 2015;154:351–7. 26536871 10.1007/s10549-015-3616-8 PMC4971774

[45] Chen X Ye G Zhang C. Superior outcome after neoadjuvant chemotherapy with docetaxel, anthracycline, and cyclophosphamide versus docetaxel plus cyclophosphamide: results from the NATT trial in triple negative or HER2 positive breast cancer. Breast Cancer Res Treat. 2013;142:549–58. 24292815 10.1007/s10549-013-2761-1

[46] Schneeweiss A Möbus V Tesch H. Intense dose-dense epirubicin, paclitaxel, cyclophosphamide versus weekly paclitaxel, liposomal doxorubicin (plus carboplatin in triple-negative breast cancer) for neoadjuvant treatment of high-risk early breast cancer (GeparOcto—GBG 84): a randomised phase III trial. Eur J Cancer. 2019;106:181–92. 30528802 10.1016/j.ejca.2018.10.015

[47] Schneeweiss A Michel LL Möbus V. Survival analysis of the randomised phase III GeparOcto trial comparing neoadjuvant chemotherapy of intense dose-dense epirubicin, paclitaxel, cyclophosphamide versus weekly paclitaxel, liposomal doxorubicin (plus carboplatin in triple-negative breast cancer) for patients with high-risk early breast cancer. Eur J Cancer. 2022;160:100–11. 34801353 10.1016/j.ejca.2021.10.011

[48] Enriquez D Nieto NP Fuentes HA Guerra H Ruiz Mendoza RE Gomez HL. Improving pathological response in locally advanced triple negative breast cancer: comparison between CbD and AC-T regimens. J Clin Oncol. 2022;35:585.

[49] Chen L Li H Zhang H. Camrelizumab vs placebo in combination with chemotherapy as neoadjuvant treatment in patients with early or locally advanced triple-negative breast cancer. JAMA. 2025;333:673. 39671272 10.1001/jama.2024.23560 PMC11862970

[50] von Minckwitz G Loibl S Untch M. Survival after neoadjuvant chemotherapy with or without bevacizumab or everolimus for HER2-negative primary breast cancer (GBG 44–GeparQuinto). Ann Oncol. 2014;25:2363–72. 25223482 10.1093/annonc/mdu455

[51] Ando M Yamauchi H Aogi K. Randomized phase II study of weekly paclitaxel with and without carboplatin followed by cyclophosphamide/epirubicin/5-fluorouracil as neoadjuvant chemotherapy for stage II/IIIA breast cancer without HER2 overexpression. Breast Cancer Res Treat. 2014;145:401–9. 24728578 10.1007/s10549-014-2947-1

[52] Iwase M Ando M Aogi K. Long-term survival analysis of addition of carboplatin to neoadjuvant chemotherapy in HER2-negative breast cancer. Breast Cancer Res Treat. 2020;180:687–94. 32140811 10.1007/s10549-020-05580-y

[53] Gonzalez-Angulo AM Akcakanat A Liu S. Open-label randomized clinical trial of standard neoadjuvant chemotherapy with paclitaxel followed by FEC versus the combination of paclitaxel and everolimus followed by FEC in women with triple receptor-negative breast cancer. Ann Oncol. 2014;25:1122–7. 24669015 10.1093/annonc/mdu124 PMC4037860

[54] Chen Z Xu L Shi W. Trends of female and male breast cancer incidence at the global, regional, and national levels, 1990–2017. Breast Cancer Res Treat. 2020;180:481–90. 32056055 10.1007/s10549-020-05561-1

[55] Guo Y Zhang H Yuan L. Machine learning and new insights for breast cancer diagnosis. J Int Med Res. 2024;52:3000605241237867. 38663911 10.1177/03000605241237867 PMC11047257

[56] Liu L Chen Z Shi W Liu H Pang W. Breast cancer survival prediction using seven prognostic biomarker genes. Oncol Lett. 2019;18:2907–16. 31452771 10.3892/ol.2019.10635 PMC6676410

[57] Yin L Duan J-J Bian X-W Yu S-C. Triple-negative breast cancer molecular subtyping and treatment progress. Breast Cancer Res. 2020;22:1–13. 10.1186/s13058-020-01296-5 PMC7285581 32517735

[58] Chaudhary LN Wilkinson KH Kong A. Triple-negative breast cancer: who should receive neoadjuvant chemotherapy? Surg Oncol Clin N Am. 2018;27:141–53. 29132557 10.1016/j.soc.2017.08.004

[59] Biswas T Efird JT Prasad S Jindal C Walker PR. The survival benefit of neoadjuvant chemotherapy and pCR among patients with advanced stage triple negative breast cancer. Oncotarget. 2017;8:112712–9. 29348858 10.18632/oncotarget.22521 PMC5762543

[60] Xu X Persson H Richardson D. Molecular pharmacology of the interaction of anthracyclines with iron. Mol Pharmacol. 2005;68:261–71. 15883202 10.1124/mol.105.013383

[61] Lorusso V Manzione L Silvestris N. Role of liposomal anthracyclines in breast cancer. Ann Oncol. 2007;18:vi70–3. 17591837 10.1093/annonc/mdm229

[62] Lim PT Goh BH Lee W-L. Taxol: mechanisms of action against cancer, an update with current research. In: Paclitaxel. Elsevier; 2022:47–71.

[63] Edwardson D Chewchuk S Parissenti AM. Resistance to anthracyclines and taxanes in breast cancer. In Breast Cancer Metastasis and Drug Resistance: Progress and Prospects. Springer; 2012:27–247.

[64] Zhang C Xu C Gao X Yao Q. Platinum-based drugs for cancer therapy and anti-tumor strategies. Theranostics. 2022;12:2115–32. 35265202 10.7150/thno.69424 PMC8899578

[65] Farrell N. Multi-platinum anti-cancer agents. Substitution-inert compounds for tumor selectivity and new targets. Chem Soc Rev. 2015;44:8773–85. 25951946 10.1039/c5cs00201j

[66] Yi M Jiao D Xu H. Biomarkers for predicting efficacy of PD-1/PD-L1 inhibitors. Mol Cancer. 2018;17:1–14. 30139382 10.1186/s12943-018-0864-3 PMC6107958

[67] Liu J Chen Z Li Y Zhao W Wu J Zhang Z. PD-1/PD-L1 checkpoint inhibitors in tumor immunotherapy. Front Pharmacol. 2021;12:731798. 34539412 10.3389/fphar.2021.731798 PMC8440961

[68] Liang SC Latchman YE Buhlmann JE. Regulation of PD‐1, PD‐L1, and PD‐L2 expression during normal and autoimmune responses. Eur J Immunol. 2003;33:2706–16. 14515254 10.1002/eji.200324228

[69] Ghiotto M Gauthier L Serriari N. PD-L1 and PD-L2 differ in their molecular mechanisms of interaction with PD-1. Int Immunol. 2010;22:651–60. 20587542 10.1093/intimm/dxq049 PMC3168865

[70] Mutebi M Anderson BO Duggan C. Breast cancer treatment: a phased approach to implementation. Cancer. 2020;126:2365–78. 32348571 10.1002/cncr.32910

[71] Dale DC. Colony-stimulating factors for the management of neutropenia in cancer patients. Drugs. 2002;62:1–15. 10.2165/00003495-200262001-00001 12479591

[72] Hesketh PJ Kris MG Basch E. Antiemetics: American Society of Clinical Oncology clinical practice guideline update. J Clin Oncol. 2017;35:3240–61. 28759346 10.1200/JCO.2017.74.4789

[73] Winer EP Lipatov O Im S-A. Pembrolizumab versus investigator-choice chemotherapy for metastatic triple-negative breast cancer (KEYNOTE-119): a randomised, open-label, phase 3 trial. Lancet Oncol. 2021;22:499–511. 33676601 10.1016/S1470-2045(20)30754-3

[74] Gray R Bradley R Braybrooke J. Increasing the dose intensity of chemotherapy by more frequent administration or sequential scheduling: a patient-level meta-analysis of 37 298 women with early breast cancer in 26 randomised trials. Lancet. 2019;393:1440–52. 30739743 10.1016/S0140-6736(18)33137-4 PMC6451189

