# Supplementary material for: Comparing efficacy of neoadjuvant therapy of triple-negative breast cancer: A Bayesian network meta-regression analysis
Source: Medicine (Baltimore). 2026 Jan 9;105(2):e46962. doi: 10.1097/MD.0000000000046962 (PMC12794967; doi:10.1097/MD.0000000000046962)

Table S1. Matrix of pairwise comparisons of regimens of PCR rate (shown as odds ratio and 95% confidence intervals)

TPtEve	0.40 (0.06, 2.90)	0.10 (0.02, 0.61)	0.18 (0.06, 0.61)	0.18 (0.03, 1.08)	0.17 (0.02, 1.19)	0.15 (0.02, 0.93)	0.14 (0.02, 0.84)	0.14 (0.02, 0.91)	0.12 (0.01, 0.97)	0.11 (0.02, 0.60)	0.11 (0.02, 0.64)	0.09 (0.01, 0.76)	0.09 (0.02, 0.44)	0.25 (0.02,2.96)	0.02 (0.00,0.31)	0.01 (0.00,0.34)
2.47 (0.35, 17.74)	ATPtBev	0.24 (0.04, 1.39)	0.46 (0.10, 2.18)	0.44 (0.08, 2.45)	0.41 (0.06, 2.72)	0.37 (0.06, 2.11)	0.34 (0.06, 1.90)	0.34 (0.06, 2.06)	0.29 (0.04, 2.24)	0.27 (0.04, 1.96)	0.27 (0.05, 1.45)	0.22 (0.03, 1.74)	0.22 (0.03, 1.45)	0.48 (0.16,1.47)	0.04 (0.00,0.73)	0.04 (0.00,0.80)
10.21 (1.64, 63.70)	4.13 (0.72, 23.60)	ATPtPD1	1.89 (0.47, 7.51)	1.83 (0.39, 8.58)	1.69 (0.81, 3.53)	1.52 (0.50, 4.63)	1.41 (0.30, 6.67)	1.39 (0.27, 7.31)	1.18 (0.40, 3.51)	1.13 (0.18, 7.04)	1.09 (0.24, 5.07)	0.91 (0.13, 6.29)	0.89 (0.15, 5.17)	0.18 (0.05,0.67)	0.18 (0.01,2.74)	0.15 (0.01,3.02)
5.42 (1.63, 17.99)	2.19 (0.46, 10.43)	0.53 (0.13, 2.11)	TPt	0.97 (0.26, 3.69)	0.89 (0.19, 4.29)	0.81 (0.20, 3.21)	0.75 (0.20, 2.87)	0.74 (0.17, 3.20)	0.63 (0.11, 3.64)	0.60 (0.18, 1.99)	0.58 (0.15, 2.18)	0.48 (0.08, 2.83)	0.47 (0.16, 1.40)	0.52 (0.15,1.77)	0.10 (0.01,1.30)	0.08 (0.01,1.45)
5.57 (0.93, 33.51)	2.25 (0.41, 12.39)	0.55 (0.12, 2.55)	1.03 (0.27, 3.90)	ATPtPDL1	0.92 (0.17, 5.09)	0.83 (0.18, 3.88)	0.77 (0.17, 3.49)	0.76 (0.15, 3.83)	0.65 (0.10, 4.26)	0.61 (0.10, 3.71)	0.60 (0.13, 2.65)	0.50 (0.07, 3.31)	0.49 (0.09, 2.71)	0.43 (0.11,1.65)	0.10 (0.01,1.46)	0.08 (0.01,1.61)
6.05 (0.84, 43.59)	2.45 (0.37, 16.25)	0.59 (0.28, 1.24)	1.12 (0.23, 5.36)	1.09 (0.20, 6.01)	ATPtPARPi	0.90 (0.24, 3.43)	0.84 (0.15, 4.67)	0.83 (0.13, 5.07)	0.70 (0.19, 2.61)	0.67 (0.09, 4.82)	0.65 (0.12, 3.56)	0.54 (0.07, 4.27)	0.53 (0.08, 3.56)	0.80 (0.17,3.88)	0.11 (0.01,1.79)	0.09 (0.01,1.96)
6.72 (1.08, 41.96)	2.72 (0.47, 15.55)	0.66 (0.22, 2.01)	1.24 (0.31, 4.95)	1.21 (0.26, 5.65)	1.11 (0.29, 4.23)	ATPDL1	0.93 (0.20, 4.39)	0.92 (0.17, 4.82)	0.78 (0.16, 3.70)	0.74 (0.12, 4.64)	0.72 (0.16, 3.34)	0.60 (0.09, 4.14)	0.59 (0.10, 3.40)	0.66 (0.23,1.89)	0.12 (0.01,1.80)	0.10 (0.01,1.99)
7.22 (1.19, 43.79)	2.92 (0.53, 16.20)	0.71 (0.15, 3.34)	1.33 (0.35, 5.11)	1.30 (0.29, 5.86)	1.19 (0.21, 6.66)	1.07 (0.23, 5.07)	ATBev	0.99 (0.19, 5.01)	0.84 (0.13, 5.57)	0.80 (0.13, 4.84)	0.77 (0.17, 3.47)	0.64 (0.10, 4.33)	0.63 (0.11, 3.55)	0.51 (0.18,1.50)	0.13 (0.01,1.90)	0.10 (0.01,2.10)
7.33 (1.10, 48.74)	2.96 (0.48, 18.11)	0.72 (0.14, 3.77)	1.35 (0.31, 5.86)	1.32 (0.26, 6.64)	1.21 (0.20, 7.44)	1.09 (0.21, 5.73)	1.01 (0.20, 5.16)	ATX	0.85 (0.12, 6.17)	0.81 (0.12, 5.39)	0.79 (0.16, 3.93)	0.65 (0.09, 4.80)	0.64 (0.10, 3.97)	0.69 (0.20,2.40)	0.13 (0.01,2.05)	0.11 (0.01,2.26)
8.63 (1.03, 72.57)	3.49 (0.45, 27.23)	0.85 (0.28, 2.51)	1.59 (0.27, 9.25)	1.55 (0.23, 10.23)	1.43 (0.38, 5.31)	1.28 (0.27, 6.09)	1.19 (0.18, 7.95)	1.18 (0.16, 8.55)	ATEve	0.95 (0.11, 8.02)	0.92 (0.14, 6.06)	0.77 (0.08, 7.06)	0.75 (0.10, 5.95)	0.61 (0.13,2.92)	0.15 (0.01,2.86)	0.13 (0.01,3.09)
9.06 (1.66, 49.59)	3.66 (0.51, 26.29)	0.89 (0.14, 5.55)	1.67 (0.50, 5.57)	1.63 (0.27, 9.80)	1.50 (0.21, 10.80)	1.35 (0.22, 8.42)	1.25 (0.21, 7.62)	1.24 (0.19, 8.23)	1.05 (0.12, 8.85)	TPtPDL1	0.97 (0.16, 5.81)	0.81 (0.09, 6.86)	0.79 (0.16, 4.00)	0.81 (0.21,3.14)	0.16 (0.01,2.83)	0.13 (0.01,3.08)
9.33 (1.56, 55.68)	3.77 (0.69, 20.58)	0.91 (0.20, 4.24)	1.72 (0.46, 6.47)	1.67 (0.38, 7.43)	1.54 (0.28, 8.46)	1.39 (0.30, 6.43)	1.29 (0.29, 5.78)	1.27 (0.25, 6.36)	1.08 (0.16, 7.09)	1.03 (0.17, 6.16)	ATPD1	0.83 (0.12, 5.51)	0.82 (0.15, 4.51)	1.25 (0.27,5.80)	0.17 (0.01,2.43)	0.14 (0.01,2.69)
11.25 (1.32, 95.81)	4.55 (0.58, 35.96)	1.10 (0.16, 7.64)	2.08 (0.35, 12.24)	2.02 (0.30, 13.53)	1.86 (0.23, 14.77)	1.67 (0.24, 11.60)	1.56 (0.23, 10.51)	1.54 (0.21, 11.30)	1.30 (0.14, 12.02)	1.24 (0.15, 10.59)	1.21 (0.18, 8.02)	ATPt	0.98 (0.12, 7.86)	0.74 (0.11,4.87)	0.20 (0.01,3.76)	0.16 (0.01,4.07)
11.44 (2.27, 57.62)	4.62 (0.69, 30.91)	1.12 (0.19, 6.48)	2.11 (0.71, 6.24)	2.05 (0.37, 11.44)	1.89 (0.28, 12.70)	1.70 (0.29, 9.85)	1.58 (0.28, 8.89)	1.56 (0.25, 9.65)	1.33 (0.17, 10.46)	1.26 (0.25, 6.37)	1.23 (0.22, 6.77)	1.02 (0.13, 8.12)	ATGem	0.73 (0.24,2.23)	0.20 (0.01,3.40)	0.17 (0.01,3.72)
4.00 (0.34, 47.37)	2.09 (0.68, 6.42)	5.68 (1.49, 21.62)	1.92 (0.57, 6.51)	2.32 (0.61, 8.90)	1.25 (0.26, 6.06)	1.51 (0.53, 4.29)	1.95 (0.67, 5.69)	1.46 (0.42, 5.09)	1.63 (0.34, 7.77)	1.23 (0.32, 4.75)	0.80 (0.17, 3.73)	1.34 (0.21, 8.81)	1.38 (0.45, 4.23)	AT	2.24 (0.47,10.66)	2.53 (0.87,7.29)
56.25 (3.21, 986.81)	22.73 (1.37, 377.52)	5.51 (0.36, 83.16)	10.39 (0.77, 139.99)	10.10 (0.69, 148.73)	9.29 (0.56, 154.84)	8.37 (0.55, 126.30)	7.79 (0.53, 115.27)	7.67 (0.49, 120.94)	6.52 (0.35, 121.37)	6.21 (0.35, 109.04)	6.03 (0.41, 88.33)	5.00 (0.27, 93.89)	4.92 (0.29, 82.31)	0.45 (0.09,2.12)	T	0.82 (0.22,3.04)
68.85 (2.95, 1609.09)	27.82 (1.25, 618.71)	6.74 (0.33, 137.56)	12.72 (0.69, 234.30)	12.36 (0.62, 246.62)	11.38 (0.51, 253.69)	10.24 (0.50, 208.92)	9.53 (0.48, 191.05)	9.39 (0.44, 199.20)	7.98 (0.32, 196.86)	7.60 (0.32, 177.78)	7.38 (0.37, 146.55)	6.12 (0.25, 152.17)	6.02 (0.27, 134.80)	0.40 (0.14,1.14)	1.22 (0.33,4.55)	TPARPi

Abbreviations: PCR, pathological complete response; AT, containing anthracyclines and taxanes; ATBev, containing anthracyclines, taxanes and bevacizumab; ATEve, containing anthracyclines, taxanes and everolimus; ATGem, containing anthracyclines, taxanes and gemcitabine; ATPD1, containing anthracyclines, taxanes and PD-1 inhibitor; ATPDL1, containing anthracyclines, taxanes and PD-L1 inhibitor; ATPt, containing anthracyclines, taxanes and platinum; ATPtBev, containing anthracyclines, taxanes, platinum and bevacizumab; ATPtPARPi, containing anthracyclines, taxanes, platinum and PARPi; ATPtPD1, containing anthracyclines, taxanes, platinum and PD-1 inhibitor; ATPtPDL1, containing anthracyclines, taxanes, platinum and PD-L1 inhibitor; ATX, containing anthracyclines, taxanes and capacitabine; T, containing taxanes only(T); TPARPi, containing taxanes and PARPi; TPt, containing taxanes and platinum; TPtEve, containing taxanes, platinum and everolimus; TPtPDL1, containing taxanes, platinum and PD-L1 inhibitor.

Table S2A. Matrix of pairwise comparisons of regimens on 12 month events free survival (shown as odds ratio and 95% confidence intervals)

TPtEve	0.67 (0.04,10.25)	0.56 (0.05,6.51)	0.55 (0.05,6.14)	0.49 (0.07,3.59)	0.34 (0.02,5.79)	0.58 (0.18,1.87)	0.13 (0.01,1.79)
1.49 (0.10,22.84)	ATGem	0.84 (0.23,3.12)	0.82 (0.23,2.87)	0.73 (0.11,4.71)	0.51 (0.06,4.51)	0.42 (0.10,1.79)	0.19 (0.03,1.31)
1.78 (0.15,20.60)	1.19 (0.32,4.44)	ATPtPD1	0.97 (0.67,1.42)	0.87 (0.21,3.62)	0.61 (0.10,3.75)	0.50 (0.22,1.12)	0.22 (0.05,1.03)
1.83 (0.16,20.59)	1.23 (0.35,4.32)	1.03 (0.71,1.50)	ATPt	0.90 (0.23,3.54)	0.63 (0.11,3.71)	0.52 (0.25,1.05)	0.23 (0.05,1.01)
2.04 (0.28,14.97)	1.37 (0.21,8.83)	1.15 (0.28,4.78)	1.12 (0.28,4.41)	TPt	0.70 (0.09,5.22)	0.83 (0.04,18.69)	0.26 (0.04,1.48)
2.93 (0.17,49.60)	1.96 (0.22,17.34)	1.64 (0.27,10.14)	1.60 (0.27,9.47)	1.43 (0.19,10.70)	ATPD1	0.83 (0.16,4.22)	0.37 (0.05,2.97)
1.73 (0.54,5.63)	2.38 (0.56,10.09)	1.99 (0.89,4.46)	1.94 (0.95,3.94)	1.21 (0.05,27.39)	1.21 (0.24,6.19)	AT	0.45 (0.12,1.64)
7.94 (0.56,112.71)	5.32 (0.76,37.17)	4.46 (0.97,20.57)	4.34 (0.99,19.06)	3.89 (0.67,22.42)	2.71 (0.34,21.84)	2.24 (0.61,8.21)	T

Abbreviations: AT, containing anthracyclines and taxanes; ATGem, containing anthracyclines, taxanes and gemcitabine; ATPD1, containing anthracyclines, taxanes and PD-1 inhibitor; ATPt, containing anthracyclines, taxanes and platinum; ATPtPD1, containing anthracyclines, taxanes, platinum and PD-1 inhibitor; T, containing taxanes only(T); TPt, containing taxanes and platinum; TPtEve, containing taxanes, platinum and everolimus;

Table S2B. Matrix of pairwise comparisons of regimens on 18 month events free survival (shown as odds ratio and 95% confidence intervals)

ATPtPD1	1.12 (0.21,5.99)	0.76 (0.23,2.44)	0.69 (0.51,0.94)	0.65 (0.30,1.45)	0.54 (0.14,2.07)	0.44 (0.24,0.80)	0.10 (0.03,0.40)
0.89 (0.17,4.79)	TPtEve	0.68 (0.20,2.25)	0.62 (0.12,3.24)	0.58 (0.10,3.57)	0.48 (0.07,3.47)	0.58 (0.21,1.58)	0.09 (0.01,0.67)
1.32 (0.41,4.26)	1.48 (0.44,4.93)	TPt	0.92 (0.30,2.84)	0.86 (0.22,3.33)	0.71 (0.15,3.41)	1.00 (0.10,9.99)	0.14 (0.03,0.66)
1.44 (1.07,1.95)	1.61 (0.31,8.42)	1.09 (0.35,3.38)	ATPt	0.94 (0.45,1.97)	0.78 (0.21,2.89)	0.63 (0.37,1.07)	0.15 (0.04,0.56)
1.53 (0.69,3.39)	1.71 (0.28,10.44)	1.16 (0.30,4.46)	1.06 (0.51,2.21)	ATGem	0.83 (0.18,3.72)	0.67 (0.27,1.66)	0.16 (0.04,0.72)
1.85 (0.48,7.12)	2.07 (0.29,14.90)	1.40 (0.29,6.69)	1.28 (0.35,4.77)	1.21 (0.27,5.45)	ATPD1	0.81 (0.24,2.70)	0.19 (0.04,1.05)
2.28 (1.24,4.19)	1.73 (0.63,4.70)	1.00 (0.10,9.92)	1.58 (0.93,2.68)	1.49 (0.60,3.69)	1.23 (0.37,4.10)	AT	0.24 (0.07,0.79)
9.64 (2.50,37.16)	10.78 (1.49,77.71)	7.29 (1.52,34.91)	6.68 (1.79,24.91)	6.30 (1.40,28.44)	5.20 (0.95,28.52)	4.22 (1.27,14.09)	T

Abbreviations: AT, containing anthracyclines and taxanes; ATGem, containing anthracyclines, taxanes and gemcitabine; ATPD1, containing anthracyclines, taxanes and PD-1 inhibitor; ATPt, containing anthracyclines, taxanes and platinum; ATPtPD1, containing anthracyclines, taxanes, platinum and PD-1 inhibitor; T, containing taxanes only(T); TPt, containing taxanes and platinum; TPtEve, containing taxanes, platinum and everolimus;

Table S2C. Matrix of pairwise comparisons of regimens on 24 month events free survival (shown as odds ratio and 95% confidence intervals)

TPtEve	0.51 (0.09,2.92)	0.45 (0.14,1.45)	0.38 (0.05,2.68)	0.33 (0.06,1.84)	0.31 (0.05,2.02)	0.46 (0.18,1.17)	0.04 (0.01,0.30)
1.95 (0.34,11.11)	ATPtPD1	0.89 (0.24,3.25)	0.74 (0.16,3.49)	0.65 (0.41,1.03)	0.60 (0.24,1.51)	0.41 (0.17,1.00)	0.08 (0.02,0.40)
2.20 (0.69,7.00)	1.13 (0.31,4.13)	TPt	0.84 (0.17,4.03)	0.74 (0.21,2.57)	0.67 (0.15,2.99)	1.25 (0.15,10.50)	0.09 (0.02,0.46)
2.63 (0.37,18.52)	1.35 (0.29,6.35)	1.20 (0.25,5.76)	ATPD1	0.88 (0.19,3.98)	0.81 (0.15,4.46)	0.56 (0.16,1.98)	0.11 (0.02,0.68)
2.99 (0.54,16.46)	1.53 (0.97,2.43)	1.36 (0.39,4.76)	1.14 (0.25,5.14)	ATPt	0.92 (0.41,2.05)	0.63 (0.28,1.43)	0.12 (0.03,0.58)
3.27 (0.50,21.55)	1.67 (0.66,4.24)	1.49 (0.33,6.59)	1.24 (0.22,6.88)	1.09 (0.49,2.45)	ATGem	0.69 (0.22,2.17)	0.14 (0.02,0.78)

2.15 (0.85,5.43)	2.43 (1.01,5.89)	0.80 (0.10,6.73)	1.80 (0.51,6.41)	1.58 (0.70,3.57)	1.45 (0.46,4.56)	AT	0.20 (0.05,0.73)
24.09 (3.32,174.56)	12.35 (2.53,60.27)	10.95 (2.19,54.62)	9.16 (1.47,56.96)	8.05 (1.72,37.79)	7.37 (1.29,42.17)	5.09 (1.37,18.94)	T

Abbreviations: AT, containing anthracyclines and taxanes; ATGem, containing anthracyclines, taxanes and gemcitabine; ATPD1, containing anthracyclines, taxanes and PD-1 inhibitor; ATPt, containing anthracyclines, taxanes and platinum; ATPtPD1, containing anthracyclines, taxanes, platinum and PD-1 inhibitor; T, containing taxanes only(T); TPt, containing taxanes and platinum; TPtEve, containing taxanes, platinum and everolimus;

Table S2D. Matrix of pairwise comparisons of regimens on 30 month events free survival (shown as odds ratio and 95% confidence intervals)

ATPtPD1	1.17 (0.22,6.20)	0.66 (0.19,2.29)	0.64 (0.39,1.04)	0.60 (0.13,2.77)	0.58 (0.23,1.47)	0.31 (0.14,0.71)	0.06 (0.01,0.29)
0.86 (0.16,4.54)	TPtEve	0.56 (0.19,1.69)	0.55 (0.11,2.75)	0.51 (0.08,3.49)	0.50 (0.08,3.00)	0.47 (0.19,1.17)	0.05 (0.01,0.37)
1.53 (0.44,5.34)	1.78 (0.59,5.37)	TPt	0.98 (0.30,3.18)	0.91 (0.19,4.39)	0.89 (0.21,3.67)	2.00 (0.29,13.99)	0.09 (0.02,0.47)
1.56 (0.96,2.53)	1.83 (0.36,9.18)	1.02 (0.31,3.33)	ATPt	0.93 (0.21,4.07)	0.91 (0.41,2.00)	0.49 (0.24,0.99)	0.10 (0.02,0.43)
1.67 (0.36,7.74)	1.95 (0.29,13.30)	1.10 (0.23,5.27)	1.07 (0.25,4.66)	ATPD1	0.97 (0.18,5.16)	0.52 (0.14,1.89)	0.10 (0.02,0.65)
1.72 (0.68,4.34)	2.01 (0.33,12.14)	1.13 (0.27,4.67)	1.10 (0.50,2.42)	1.03 (0.19,5.47)	ATGem	0.54 (0.19,1.55)	0.11 (0.02,0.58)
3.21 (1.40,7.38)	2.11 (0.86,5.17)	0.50 (0.07,3.49)	2.06 (1.01,4.19)	1.92 (0.53,6.97)	1.87 (0.65,5.40)	AT	0.20 (0.05,0.75)
16.35 (3.39,78.78)	19.11 (2.72,134.32)	10.72 (2.14,53.56)	10.47 (2.31,47.51)	9.79 (1.53,62.55)	9.50 (1.73,52.33)	5.09 (1.34,19.33)	T

Abbreviations: AT, containing anthracyclines and taxanes; ATGem, containing anthracyclines, taxanes and gemcitabine; ATPD1, containing anthracyclines, taxanes and PD-1 inhibitor; ATPt, containing anthracyclines, taxanes and platinum; ATPtPD1, containing anthracyclines, taxanes, platinum and PD-1 inhibitor; T, containing taxanes only(T); TPt, containing taxanes and platinum; TPtEve, containing taxanes, platinum and everolimus;

Table S2E. Matrix of pairwise comparisons of regimens on 36 month events free survival (shown as odds ratio and 95% confidence intervals)

ATPtPD1	0.69 (0.08,6.16)	0.60 (0.21,1.69)	0.57 (0.12,2.65)	0.52 (0.07,3.84)	0.47 (0.09,2.60)	0.24 (0.06,0.87)
1.44 (0.16,12.76)	TPtEve	0.86 (0.13,5.88)	0.82 (0.09,7.62)	0.74 (0.07,7.48)	0.68 (0.17,2.65)	0.50 (0.17,1.46)
1.67 (0.59,4.71)	1.16 (0.17,7.90)	ATPt	0.95 (0.31,2.95)	0.86 (0.15,4.80)	0.79 (0.20,3.04)	0.39 (0.18,0.86)
1.75 (0.38,8.12)	1.22 (0.13,11.27)	1.05 (0.34,3.25)	ATGem	0.90 (0.12,7.06)	0.83 (0.14,4.81)	0.41 (0.10,1.64)
1.94 (0.26,14.44)	1.35 (0.13,13.58)	1.16 (0.21,6.48)	1.11 (0.14,8.66)	ATPD1	0.91 (0.14,5.92)	0.46 (0.10,2.11)
2.12 (0.39,11.67)	1.47 (0.38,5.75)	1.27 (0.33,4.92)	1.21 (0.21,7.06)	1.09 (0.17,7.07)	TPt	1.54 (0.17,14.09)
4.23 (1.15,15.57)	2.00 (0.68,5.83)	2.54 (1.16,5.57)	2.42 (0.61,9.57)	2.18 (0.47,10.06)	0.65 (0.07,5.95)	AT

Abbreviations: AT, containing anthracyclines and taxanes; ATGem, containing anthracyclines, taxanes and gemcitabine; ATPD1, containing anthracyclines, taxanes and PD-1 inhibitor; ATPt, containing anthracyclines, taxanes and platinum; ATPtPD1, containing anthracyclines, taxanes, platinum and PD-1 inhibitor; TPt, containing taxanes and platinum; TPtEve, containing taxanes, platinum and everolimus;

Table S2F. Matrix of pairwise comparisons of regimens on 42 month events free survival (shown as odds ratio and 95% confidence intervals)

ATPtPD1	0.83 (0.11,6.40)	0.58 (0.23,1.50)	0.57 (0.14,2.32)	0.43 (0.09,2.17)	0.22 (0.06,0.73)
1.21 (0.16,9.39)	TPtEve	0.70 (0.11,4.33)	0.69 (0.08,5.57)	0.53 (0.15,1.87)	0.50 (0.18,1.39)
1.72 (0.67,4.44)	1.42 (0.23,8.72)	ATPt	0.98 (0.34,2.76)	0.75 (0.20,2.74)	0.37 (0.18,0.79)
1.76 (0.43,7.21)	1.46 (0.18,11.80)	1.02 (0.36,2.90)	ATGem	0.77 (0.15,4.05)	0.38 (0.11,1.38)
2.30 (0.46,11.49)	1.90 (0.53,6.74)	1.34 (0.36,4.90)	1.30 (0.25,6.89)	TPt	1.46 (0.17,12.47)
4.62 (1.38,15.50)	2.01 (0.72,5.62)	2.68 (1.27,5.69)	2.62 (0.73,9.46)	0.68 (0.08,5.82)	AT

Abbreviations: AT, containing anthracyclines and taxanes; ATGem, containing anthracyclines, taxanes and gemcitabine; ATPt, containing anthracyclines, taxanes and platinum; ATPtPD1, containing anthracyclines, taxanes, platinum and PD-1 inhibitor; TPt, containing taxanes and platinum; TPtEve, containing taxanes, platinum and everolimus;

Table S2G. Matrix of pairwise comparisons of regimens on 48 month events free survival (shown as odds ratio and 95% confidence intervals)

ATPtPD1	0.94 (0.15,5.70)	0.53 (0.17,1.71)	0.54 (0.25,1.16)	0.49 (0.12,2.00)	0.25 (0.09,0.68)
1.06 (0.18,6.46)	TPtEve	0.57 (0.09,3.62)	0.57 (0.11,2.94)	0.53 (0.17,1.65)	0.50 (0.20,1.28)
1.87 (0.59,5.99)	1.76 (0.28,11.19)	ATGem	1.01 (0.42,2.42)	0.93 (0.22,3.98)	0.46 (0.15,1.39)
1.85 (0.86,3.99)	1.74 (0.34,8.90)	0.99 (0.41,2.37)	ATPt	0.92 (0.29,2.95)	0.46 (0.24,0.89)
2.02 (0.50,8.17)	1.90 (0.61,5.93)	1.08 (0.25,4.64)	1.09 (0.34,3.51)	TPt	1.79 (0.25,13.11)
4.04 (1.47,11.13)	2.00 (0.78,5.10)	2.16 (0.72,6.46)	2.18 (1.12,4.23)	0.56 (0.08,4.07)	AT

Abbreviations: AT, containing anthracyclines and taxanes; ATGem, containing anthracyclines, taxanes and gemcitabine; ATPt, containing anthracyclines, taxanes and platinum; ATPtPD1, containing anthracyclines, taxanes, platinum and PD-1 inhibitor; TPt, containing taxanes and platinum; TPtEve, containing taxanes, platinum and everolimus;

Table S3A. Matrix of pairwise comparisons of regimens on 12 month overall survival (shown as odds ratio and 95% confidence intervals)

ATGem	0.30 (0.01,7.38)	0.19 (0.00,10.08)	0.19 (0.01,6.23)	0.14 (0.00,4.03)	0.02 (0.00,2.12)
3.35 (0.14,82.89)	ATPt	0.62 (0.06,6.72)	0.64 (0.16,2.51)	0.47 (0.18,1.26)	0.07 (0.00,1.94)
5.38 (0.10,292.01)	1.61 (0.15,17.33)	TPt	0.58 (0.01,57.34)	0.76 (0.06,9.94)	0.11 (0.00,4.11)
5.25 (0.16,171.74)	1.57 (0.40,6.16)	1.74 (0.02,173.23)	AT	0.74 (0.14,3.99)	0.11 (0.01,2.26)
7.10 (0.25,203.52)	2.12 (0.79,5.66)	1.32 (0.10,17.31)	1.35 (0.25,7.30)	ATPtPD1	0.15 (0.00,4.75)
47.49 (0.47,4786.21)	14.17 (0.51,389.90)	8.82 (0.24,320.11)	9.05 (0.44,185.19)	6.69 (0.21,212.19)	T

Abbreviations: AT, containing anthracyclines and taxanes; ATGem, containing anthracyclines, taxanes and gemcitabine; ATPt, containing anthracyclines, taxanes and platinum; ATPtPD1, containing anthracyclines, taxanes, platinum and PD-1 inhibitor; TPt, containing taxanes and platinum; T, containing taxanes only.

Table S3B. Matrix of pairwise comparisons of regimens on 18 month overall survival (shown as odds ratio and 95% confidence intervals)

ATPtPD1	0.76 (0.46,1.28)	0.47 (0.07,3.29)	0.36 (0.08,1.54)	0.28 (0.10,0.75)	0.02 (0.00,0.40)
1.31 (0.78,2.20)	ATPt	0.62 (0.09,4.02)	0.47 (0.12,1.83)	0.37 (0.16,0.85)	0.02 (0.00,0.50)
2.12 (0.30,14.87)	1.62 (0.25,10.60)	TPt	0.76 (0.07,7.71)	0.57 (0.02,16.50)	0.04 (0.00,1.13)
2.81 (0.65,12.16)	2.15 (0.55,8.46)	1.32 (0.13,13.50)	ATGem	0.79 (0.16,3.93)	0.05 (0.00,1.45)
3.56 (1.33,9.52)	2.72 (1.18,6.28)	1.76 (0.06,51.24)	1.27 (0.25,6.31)	AT	0.06 (0.00,1.22)
56.10 (2.49,1263.92)	42.88 (1.99,925.08)	26.40 (0.88,790.08)	19.96 (0.69,576.53)	15.76 (0.82,302.66)	T

Abbreviations: AT, containing anthracyclines and taxanes; ATGem, containing anthracyclines, taxanes and gemcitabine; ATPt, containing anthracyclines, taxanes and platinum; ATPtPD1, containing anthracyclines, taxanes, platinum and PD-1 inhibitor; TPt, containing taxanes and platinum; T, containing taxanes only.

Table S3C. Matrix of pairwise comparisons of regimens on 24 month overall survival (shown as odds ratio and 95% confidence intervals)

ATPtPD1	0.83 (0.29,2.39)	0.58 (0.07,4.64)	0.48 (0.09,2.68)	0.45 (0.11,1.84)	0.09 (0.01,0.95)
1.21 (0.42,3.48)	ATPt	0.70 (0.12,4.18)	0.58 (0.15,2.24)	0.54 (0.21,1.37)	0.10 (0.01,0.89)
1.72 (0.22,13.67)	1.42 (0.24,8.47)	TPt	0.82 (0.09,7.73)	0.65 (0.04,11.17)	0.15 (0.01,1.74)
2.08 (0.37,11.62)	1.73 (0.45,6.69)	1.21 (0.13,11.39)	ATGem	0.93 (0.18,4.83)	0.18 (0.01,2.28)
2.23 (0.54,9.14)	1.85 (0.73,4.69)	1.55 (0.09,26.75)	1.07 (0.21,5.54)	AT	0.19 (0.03,1.34)
11.70 (1.05,130.05)	9.71 (1.12,84.38)	6.82 (0.57,80.89)	5.62 (0.44,72.04)	5.25 (0.75,36.95)	T

Abbreviations: AT, containing anthracyclines and taxanes; ATGem, containing anthracyclines, taxanes and gemcitabine; ATPt, containing anthracyclines, taxanes and platinum; ATPtPD1, containing anthracyclines, taxanes, platinum and PD-1 inhibitor; TPt, containing taxanes and platinum; T, containing taxanes only.

Table S3D. Matrix of pairwise comparisons of regimens on 30 month overall survival (shown as odds ratio and 95% confidence intervals)

ATPtPD1	0.80 (0.53,1.18)	0.45 (0.18,1.13)	0.42 (0.12,1.51)	0.40 (0.21,0.79)	0.08 (0.01,0.47)
1.26 (0.85,1.87)	ATPt	0.56 (0.24,1.30)	0.53 (0.16,1.78)	0.51 (0.29,0.87)	0.10 (0.02,0.57)
2.24 (0.88,5.66)	1.78 (0.77,4.12)	ATGem	0.94 (0.21,4.12)	0.90 (0.33,2.44)	0.17 (0.02,1.22)
2.39 (0.66,8.60)	1.90 (0.56,6.42)	1.07 (0.24,4.69)	TPt	1.29 (0.16,10.36)	0.18 (0.02,1.37)
2.49 (1.27,4.87)	1.98 (1.15,3.40)	1.11 (0.41,3.02)	0.78 (0.10,6.23)	AT	0.19 (0.04,1.04)
13.05 (2.11,80.74)	10.38 (1.75,61.47)	5.84 (0.82,41.71)	5.47 (0.73,41.04)	5.25 (0.96,28.57)	T

Abbreviations: AT, containing anthracyclines and taxanes; ATGem, containing anthracyclines, taxanes and gemcitabine; ATPt, containing anthracyclines, taxanes and platinum; ATPtPD1, containing anthracyclines, taxanes, platinum and PD-1 inhibitor; TPt, containing taxanes and platinum; T, containing taxanes only.

Table S3E. Matrix of pairwise comparisons of regimens on 36 month overall survival (shown as odds ratio and 95% confidence intervals)

ATPtPD1	0.78 (0.53,1.13)	0.54 (0.17,1.74)	0.42 (0.19,0.96)	0.40 (0.22,0.75)
1.29 (0.89,1.87)	ATPt	0.70 (0.23,2.10)	0.54 (0.26,1.13)	0.52 (0.31,0.85)
1.85 (0.58,5.94)	1.44 (0.48,4.34)	TPt	0.78 (0.21,2.95)	1.70 (0.23,12.83)
2.37 (1.04,5.40)	1.84 (0.88,3.83)	1.28 (0.34,4.83)	ATGem	0.95 (0.39,2.31)
2.49 (1.34,4.65)	1.94 (1.18,3.19)	0.59 (0.08,4.42)	1.05 (0.43,2.56)	AT

Abbreviations: AT, containing anthracyclines and taxanes; ATGem, containing anthracyclines, taxanes and gemcitabine; ATPt, containing anthracyclines, taxanes and platinum; ATPtPD1, containing anthracyclines, taxanes, platinum and PD-1 inhibitor; TPt, containing taxanes and platinum.

Table S3F. Matrix of pairwise comparisons of regimens on 42 month overall survival (shown as odds ratio and 95% confidence intervals)

ATPtPD1	0.73 (0.51,1.05)	0.52 (0.24,1.12)	0.33 (0.11,1.01)	0.32 (0.17,0.58)
1.36 (0.96,1.95)	ATPt	0.71 (0.36,1.39)	0.45 (0.16,1.31)	0.43 (0.27,0.70)
1.92 (0.90,4.12)	1.41 (0.72,2.76)	ATGem	0.64 (0.18,2.24)	0.61 (0.26,1.39)
3.01 (0.99,9.16)	2.20 (0.77,6.34)	1.56 (0.45,5.48)	TPt	1.11 (0.15,8.15)
3.17 (1.74,5.78)	2.32 (1.43,3.77)	1.65 (0.72,3.78)	0.90 (0.12,6.60)	AT

Abbreviations: AT, containing anthracyclines and taxanes; ATGem, containing anthracyclines, taxanes and gemcitabine; ATPt, containing anthracyclines, taxanes and platinum; ATPtPD1, containing anthracyclines, taxanes, platinum and PD-1 inhibitor; TPt, containing taxanes and platinum.

Table S3G. Matrix of pairwise comparisons of regimens on 48 month overall survival (shown as odds ratio and 95% confidence intervals)

ATPtPD1	0.70 (0.39,1.27)	0.57 (0.22,1.52)	0.40 (0.10,1.62)	0.34 (0.13,0.90)
1.42 (0.79,2.58)	ATPt	0.82 (0.38,1.77)	0.56 (0.16,2.02)	0.48 (0.22,1.04)
1.74 (0.66,4.63)	1.23 (0.57,2.66)	ATGem	0.69 (0.15,3.07)	0.59 (0.20,1.76)
2.53 (0.62,10.36)	1.78 (0.50,6.38)	1.45 (0.33,6.46)	TPt	1.38 (0.16,12.09)
2.97 (1.12,7.91)	2.09 (0.96,4.54)	1.70 (0.57,5.10)	0.73 (0.08,6.38)	AT

Abbreviations: AT, containing anthracyclines and taxanes; ATGem, containing anthracyclines, taxanes and gemcitabine; ATPt, containing anthracyclines, taxanes and platinum; ATPtPD1, containing anthracyclines, taxanes, platinum and PD-1 inhibitor; TPt, containing taxanes and platinum.

Table S4A. Matrix of pairwise comparisons of regimens on HR of EFS (shown as hazard ratio and 95% confidence intervals).

AT	0.98 (0.7, 1.36)	1.59 (0.86, 3.07)	1.31 (0.63, 2.75)	1.53 (1.17, 2.08)	1.42 (0.88, 2.29)	1.64 (1.1, 2.53)	2.24 (1.42, 3.59)	1.31 (0.33, 5.23)	2.02 (0.39, 10.64)
1.02 (0.73, 1.44)	ATBev	1.62 (0.81, 3.42)	1.33 (0.6, 3.03)	1.56 (1.03, 2.51)	1.45 (0.89, 2.36)	1.67 (1, 2.93)	2.28 (1.31, 4.12)	1.33 (0.32, 5.56)	2.06 (0.38, 11.24)
0.63 (0.33, 1.16)	0.62 (0.29, 1.23)	ATGem	0.83 (0.31, 2.14)	0.96 (0.55, 1.69)	0.89 (0.39, 1.93)	1.04 (0.51, 2.07)	1.41 (0.71, 2.72)	0.82 (0.18, 3.75)	1.27 (0.21, 7.43)
0.76 (0.36, 1.59)	0.75 (0.33, 1.68)	1.21 (0.47, 3.27)	ATPDL1	1.17 (0.53, 2.61)	1.08 (0.45, 2.62)	1.26 (0.54, 2.95)	1.71 (0.72, 4.1)	1 (0.21, 4.84)	1.54 (0.25, 9.55)
0.65 (0.48, 0.86)	0.64 (0.4, 0.97)	1.04 (0.59, 1.83)	0.86 (0.38, 1.87)	ATPt	0.93 (0.52, 1.59)	1.08 (0.7, 1.62)	1.47 (0.99, 2.08)	0.85 (0.21, 3.48)	1.32 (0.24, 7.06)
0.71 (0.44, 1.14)	0.69 (0.42, 1.12)	1.12 (0.52, 2.54)	0.92 (0.38, 2.24)	1.08 (0.63, 1.93)	ATPtBev	1.16 (0.62, 2.22)	1.58 (0.81, 3.1)	0.92 (0.21, 3.96)	1.42 (0.26, 7.97)
0.61 (0.4, 0.91)	0.6 (0.34, 1)	0.96 (0.48, 1.98)	0.8 (0.34, 1.85)	0.93 (0.62, 1.43)	0.86 (0.45, 1.61)	ATPtPARPi	1.36 (0.78, 2.37)	0.79 (0.19, 3.36)	1.22 (0.22, 6.75)
0.45 (0.28, 0.7)	0.44 (0.24, 0.76)	0.71 (0.37, 1.42)	0.59 (0.24, 1.39)	0.68 (0.48, 1.01)	0.63 (0.32, 1.23)	0.73 (0.42, 1.29)	ATPtPD1	0.58 (0.14, 2.51)	0.9 (0.16, 5.03)
0.76 (0.19, 3.07)	0.75 (0.18, 3.11)	1.22 (0.27, 5.66)	1 (0.21, 4.87)	1.17 (0.29, 4.86)	1.08 (0.25, 4.71)	1.26 (0.3, 5.37)	1.71 (0.4, 7.39)	TPt	1.54 (0.63, 3.78)

0.49 (0.09, 2.59)	0.49 (0.09, 2.61)	0.79 (0.13, 4.66)	0.65 (0.1, 3.96)	0.76 (0.14, 4.08)	0.7 (0.13, 3.91)	0.82 (0.15, 4.5)	1.11 (0.2, 6.17)	0.65 (0.26, 1.59)	TPtEve

Abbreviations: HR, hazard ratio; EFS, events free survival; AT, containing anthracyclines and taxanes; ATBev, containing anthracyclines, taxanes and bevacizumab; ATGem, containing anthracyclines, taxanes and gemcitabine; ATPDL1, containing anthracyclines, taxanes and PD-L1 inhibitor; ATPt, containing anthracyclines, taxanes and platinum; ATPtBev, containing anthracyclines, taxanes, platinum and bevacizumab; ATPtPARPi, containing anthracyclines, taxanes, platinum and PARPi; ATPtPD1, containing anthracyclines, taxanes, platinum and PD-1 inhibitor; TPt, containing taxanes and platinum; TPtEve, containing taxanes, platinum and everolimus.

Table S4B. Matrix of pairwise comparisons of regimens on HR of EFS after Bayesian network meta-regression (shown as hazard ratio and 95% confidence intervals).

AT	1.01 (0.68, 1.51)	1.63 (0.85, 3.33)	1.2 (0.52, 2.82)	1.57 (1.17, 2.22)	1.53 (0.85, 2.76)	1.66 (1.08, 2.65)	2.29 (1.4, 3.87)	1.26 (0.31, 5.15)	1.95 (0.36, 10.52)
0.99 (0.66, 1.48)	ATBev	1.61 (0.77, 3.62)	1.18 (0.44, 3.24)	1.55 (0.99, 2.6)	1.51 (0.88, 2.58)	1.64 (0.93, 3.01)	2.27 (1.23, 4.31)	1.25 (0.29, 5.47)	1.93 (0.34, 11.06)
0.61 (0.3, 1.18)	0.62 (0.28, 1.3)	ATGem	0.73 (0.24, 2.2)	0.96 (0.53, 1.76)	0.94 (0.38, 2.19)	1.02 (0.47, 2.15)	1.41 (0.67, 2.86)	0.77 (0.16, 3.69)	1.19 (0.19, 7.33)
0.84 (0.35, 1.93)	0.85 (0.31, 2.25)	1.36 (0.45, 4.26)	ATPDL1	1.31 (0.52, 3.4)	1.28 (0.41, 3.93)	1.39 (0.53, 3.69)	1.91 (0.69, 5.31)	1.05 (0.21, 5.27)	1.63 (0.25, 10.31)
0.64 (0.45, 0.85)	0.65 (0.38, 1.01)	1.04 (0.57, 1.9)	0.76 (0.29, 1.92)	ATPt	0.97 (0.51, 1.78)	1.06 (0.66, 1.65)	1.47 (0.95, 2.14)	0.8 (0.19, 3.39)	1.24 (0.22, 6.85)
0.65 (0.36, 1.18)	0.66 (0.39, 1.13)	1.07 (0.46, 2.64)	0.78 (0.25, 2.44)	1.03 (0.56, 1.98)	ATPtBev	1.09 (0.54, 2.27)	1.5 (0.72, 3.19)	0.83 (0.17, 3.92)	1.28 (0.21, 7.81)
0.6 (0.38, 0.92)	0.61 (0.33, 1.08)	0.98 (0.47, 2.12)	0.72 (0.27, 1.9)	0.94 (0.61, 1.51)	0.92 (0.44, 1.86)	ATPtPARPi	1.38 (0.75, 2.51)	0.76 (0.17, 3.3)	1.17 (0.2, 6.65)
0.44 (0.26, 0.72)	0.44 (0.23, 0.81)	0.71 (0.35, 1.5)	0.52 (0.19, 1.44)	0.68 (0.47, 1.05)	0.67 (0.31, 1.39)	0.73 (0.4, 1.34)	ATPtPD1	0.55 (0.12, 2.46)	0.85 (0.15, 4.93)
0.79 (0.19, 3.21)	0.8 (0.18, 3.5)	1.3 (0.27, 6.3)	0.95 (0.19, 4.71)	1.25 (0.3, 5.35)	1.21 (0.26, 5.72)	1.32 (0.3, 5.8)	1.83 (0.41, 8.15)	TPt	1.55 (0.62, 3.88)
0.51 (0.1, 2.75)	0.52 (0.09, 2.96)	0.84 (0.14, 5.27)	0.61 (0.1, 3.92)	0.81 (0.15, 4.55)	0.78 (0.13, 4.77)	0.85 (0.15, 4.95)	1.18 (0.2, 6.88)	0.65 (0.26, 1.62)	TPtEve

Abbreviations: HR, hazard ratio; EFS, events free survival; AT, containing anthracyclines and taxanes; ATBev, containing anthracyclines, taxanes and bevacizumab; ATGem, containing anthracyclines, taxanes and gemcitabine; ATPDL1, containing anthracyclines, taxanes and PD-L1 inhibitor; ATPt, containing anthracyclines, taxanes and platinum; ATPtBev, containing anthracyclines, taxanes, platinum and bevacizumab; ATPtPARPi, containing anthracyclines, taxanes, platinum and PARPi; ATPtPD1, containing anthracyclines, taxanes, platinum and PD-1 inhibitor; TPt, containing taxanes and platinum; TPtEve, containing taxanes, platinum and everolimus.

Table S5A. Matrix of pairwise comparisons of regimens on HR of OS (shown as hazard ratio and 95% confidence intervals).

AT	0.93 (0.36, 2.37)	1.28 (0.47, 3.74)	1.45 (0.41, 5.08)	1.36 (0.88, 2.23)	0.93 (0.36, 2.35)	1.16 (0.58, 2.37)	2.67 (1.03, 7.35)	1.04 (0.18, 6.07)
1.08 (0.42, 2.76)	ATBev	1.39 (0.35, 5.78)	1.57 (0.33, 7.43)	1.47 (0.53, 4.31)	1 (0.27, 3.75)	1.25 (0.39, 4.07)	2.88 (0.76, 11.54)	1.12 (0.15, 8.21)
0.78 (0.27, 2.13)	0.72 (0.17, 2.85)	ATGem	1.13 (0.22, 5.57)	1.06 (0.42, 2.68)	0.72 (0.17, 2.83)	0.9 (0.28, 2.84)	2.08 (0.58, 7.4)	0.8 (0.1, 6.14)
0.69 (0.2, 2.41)	0.64 (0.13, 3.02)	0.89 (0.18, 4.56)	ATPDL1	0.94 (0.25, 3.63)	0.64 (0.13, 3.04)	0.8 (0.19, 3.39)	1.85 (0.39, 9.16)	0.71 (0.08, 6.16)
0.73 (0.45, 1.14)	0.68 (0.23, 1.89)	0.94 (0.37, 2.39)	1.07 (0.28, 3.97)	ATPt	0.68 (0.23, 1.87)	0.85 (0.41, 1.7)	1.97 (0.82, 4.67)	0.76 (0.12, 4.66)
1.08 (0.42, 2.75)	1 (0.27, 3.76)	1.38 (0.35, 5.77)	1.57 (0.33, 7.41)	1.47 (0.53, 4.3)	ATPtBev	1.25 (0.39, 4.08)	2.88 (0.76, 11.53)	1.12 (0.15, 8.22)
0.86 (0.42, 1.73)	0.8 (0.25, 2.56)	1.11 (0.35, 3.63)	1.25 (0.3, 5.21)	1.18 (0.59, 2.43)	0.8 (0.25, 2.54)	ATPtPARPi	2.31 (0.77, 7.19)	0.89 (0.13, 5.96)
0.37 (0.14, 0.98)	0.35 (0.09, 1.31)	0.48 (0.14, 1.71)	0.54 (0.11, 2.58)	0.51 (0.21, 1.22)	0.35 (0.09, 1.31)	0.43 (0.14, 1.31)	ATPtPD1	0.39 (0.05, 2.86)
0.97 (0.16, 5.66)	0.9 (0.12, 6.51)	1.25 (0.16, 9.69)	1.41 (0.16, 12.24)	1.32 (0.21, 8.27)	0.9 (0.12, 6.55)	1.12 (0.17, 7.48)	2.59 (0.35, 19.56)	TPt

Abbreviations: HR, hazard ratio; OS, overall survival; AT, containing anthracyclines and taxanes; ATBev, containing anthracyclines, taxanes and bevacizumab; ATGem, containing anthracyclines, taxanes and gemcitabine; ATPDL1, containing anthracyclines, taxanes and PD-L1 inhibitor; ATPt, containing anthracyclines, taxanes and platinum; ATPtBev, containing anthracyclines, taxanes, platinum and bevacizumab; ATPtPARPi, containing anthracyclines, taxanes, platinum and PARPi; ATPtPD1, containing anthracyclines, taxanes, platinum and PD-1 inhibitor; TPt, containing taxanes and platinum.

Table S5B. Matrix of pairwise comparisons of regimens on HR of OS after Bayesian network meta-regression (shown as hazard ratio and 95% confidence intervals).

AT	1.4 (0.49, 3.75)	1.3 (0.53, 3.25)	0.87 (0.23, 3.58)	1.38 (0.93, 2.11)	1.4 (0.49, 3.72)	1.11 (0.61, 2.09)	2.71 (1.19, 6.39)	0.78 (0.13, 4.61)
0.71 (0.27, 2.02)	ATBev	0.93 (0.25, 3.78)	0.62 (0.09, 4.59)	0.98 (0.35, 3.07)	1 (0.32, 3.14)	0.79 (0.25, 2.79)	1.92 (0.55, 7.6)	0.56 (0.07, 4.88)
0.77 (0.31, 1.87)	1.08 (0.26, 3.95)	ATGem	0.67 (0.13, 3.59)	1.06 (0.48, 2.37)	1.08 (0.26, 3.94)	0.85 (0.31, 2.36)	2.08 (0.7, 6.17)	0.59 (0.08, 4.36)
1.14 (0.28, 4.42)	1.6 (0.22, 10.88)	1.49 (0.28, 7.6)	ATPDL1	1.58 (0.36, 6.57)	1.6 (0.22, 10.8)	1.27 (0.28, 5.43)	3.11 (0.6, 15.12)	0.88 (0.11, 7.2)
0.72 (0.47, 1.08)	1.02 (0.33, 2.87)	0.94 (0.42, 2.1)	0.63 (0.15, 2.75)	ATPt	1.02 (0.33, 2.85)	0.8 (0.43, 1.5)	1.96 (0.94, 4.09)	0.56 (0.09, 3.47)
0.71 (0.27, 2.03)	1 (0.32, 3.14)	0.93 (0.25, 3.78)	0.62 (0.09, 4.63)	0.98 (0.35, 3.05)	ATPtBev	0.79 (0.25, 2.8)	1.93 (0.55, 7.56)	0.56 (0.07, 4.94)
0.9 (0.48, 1.64)	1.26 (0.36, 4.07)	1.17 (0.42, 3.23)	0.79 (0.18, 3.55)	1.24 (0.67, 2.33)	1.27 (0.36, 4.07)	ATPtPARPi	2.44 (0.94, 6.42)	0.7 (0.11, 4.5)
0.37 (0.16, 0.84)	0.52 (0.13, 1.81)	0.48 (0.16, 1.42)	0.32 (0.07, 1.66)	0.51 (0.24, 1.06)	0.52 (0.13, 1.8)	0.41 (0.16, 1.07)	ATPtPD1	0.29 (0.04, 2.01)
1.29 (0.22, 7.43)	1.79 (0.2, 15.19)	1.68 (0.23, 12.15)	1.13 (0.14, 9.13)	1.79 (0.29, 10.83)	1.8 (0.2, 15.29)	1.43 (0.22, 9.05)	3.51 (0.5, 24.28)	TPt

Abbreviations: HR, hazard ratio; OS, overall survival; AT, containing anthracyclines and taxanes; ATBev, containing anthracyclines, taxanes and bevacizumab; ATGem, containing anthracyclines, taxanes and gemcitabine; ATPDL1, containing anthracyclines, taxanes and PD-L1 inhibitor; ATPt, containing anthracyclines, taxanes and platinum; ATPtBev, containing anthracyclines, taxanes, platinum and bevacizumab; ATPtPARPi, containing anthracyclines, taxanes, platinum and PARPi; ATPtPD1, containing anthracyclines, taxanes, platinum and PD-1 inhibitor; TPt, containing taxanes and platinum.

Figure S1. Summary of quality assessments using Cochrane Risk of Bias Tool 2.

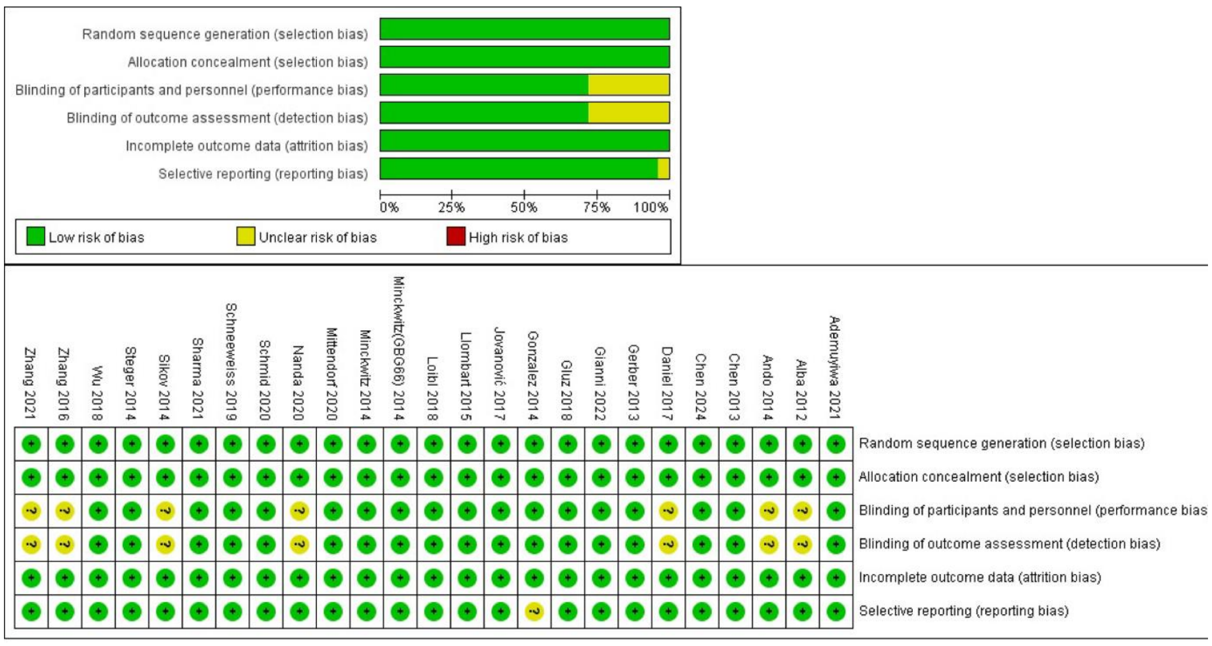

Figure S2. Convergence of the three Markov Chain Monte Carlo chains established by of the history feature for HR of events free survival. AT, containing anthracyclines and taxanes; ATBev, containing anthracyclines, taxanes and bevacizumab; ATGem, containing anthracyclines, taxanes and gemcitabine; ATPDL1, containing anthracyclines, taxanes and PD-L1 inhibitor; ATPt, containing anthracyclines, taxanes and platinum; ATPtBev, containing anthracyclines, taxanes, platinum and bevacizumab; ATPtPARPi, containing anthracyclines, taxanes, platinum and PARPi; ATPtPD1, containing anthracyclines, taxanes, platinum and PD-1 inhibitor; TPt, containing taxanes and platinum; TPtEve, containing taxanes, platinum and everolimus.

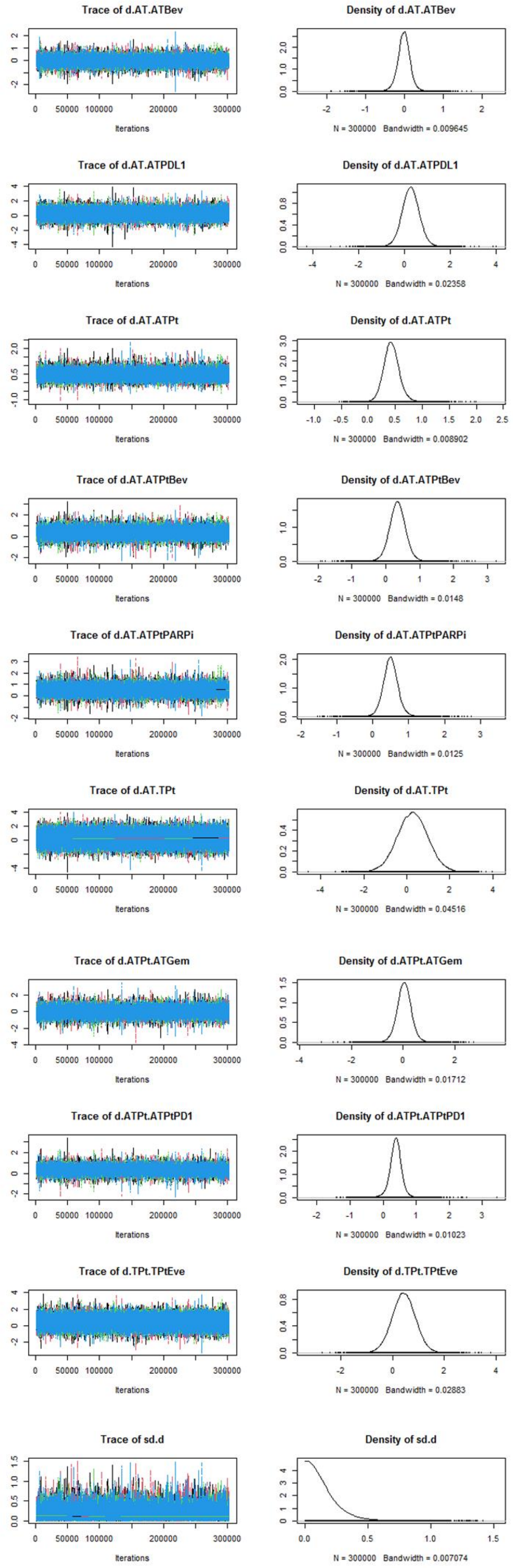

Figure S3. Convergence of the three Markov Chain Monte Carlo chains established by of the history feature for HR of events free survival after regression analysis.AT, containing anthracyclines and taxanes; ATBev, containing anthracyclines, taxanes and bevacizumab; ATGem, containing anthracyclines, taxanes and gemcitabine; ATPDL1, containing anthracyclines, taxanes and PD-L1 inhibitor; ATPt, containing anthracyclines, taxanes and platinum; ATPtBev, containing anthracyclines, taxanes, platinum and bevacizumab; ATPtPARPi, containing anthracyclines, taxanes, platinum and PARPi; ATPtPD1, containing anthracyclines, taxanes, platinum and PD-1 inhibitor; TPt, containing taxanes and platinum; TPtEve, containing taxanes, platinum and everolimus.

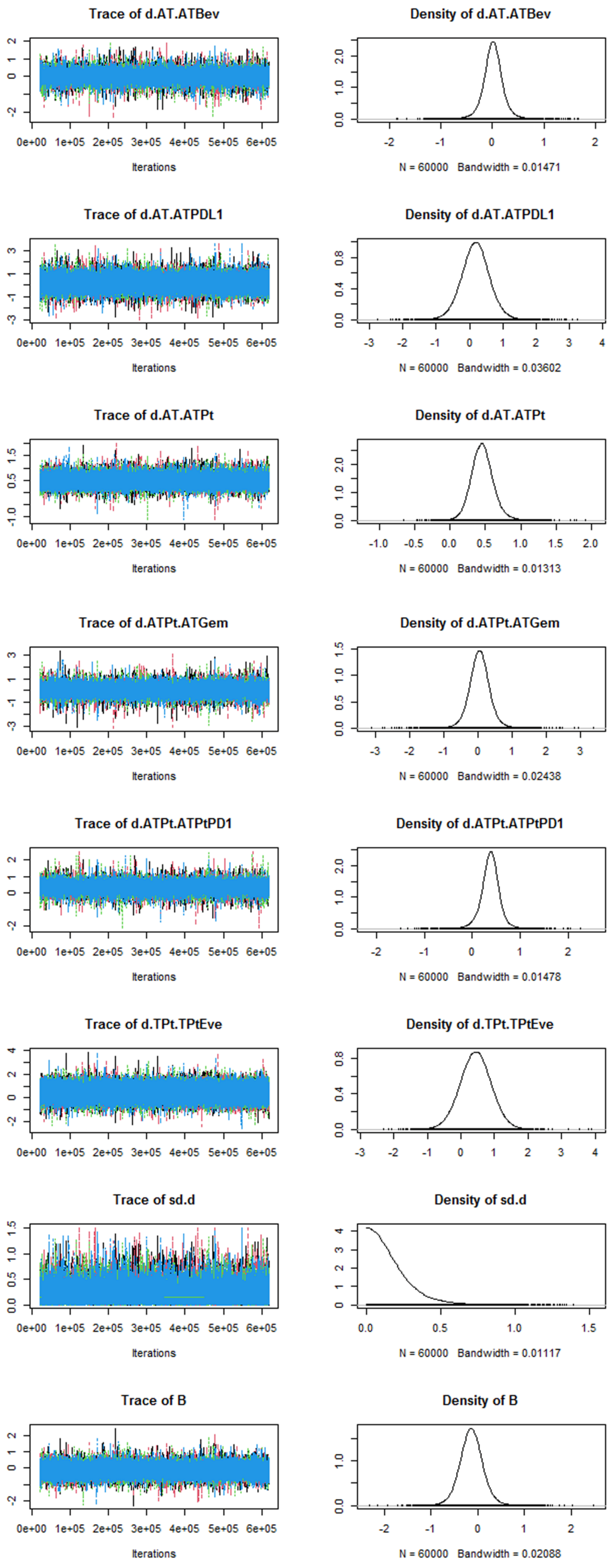

Figure S4. Convergence of the three Markov Chain Monte Carlo chains established by of the history feature for HR of overall survival. AT, containing anthracyclines and taxanes; ATBev, containing anthracyclines, taxanes and bevacizumab; ATGem, containing anthracyclines, taxanes and gemcitabine; ATPDL1, containing anthracyclines, taxanes and PD-L1 inhibitor; ATPt, containing anthracyclines, taxanes and platinum; ATPtBev, containing anthracyclines, taxanes, platinum and bevacizumab; ATPtPARPi, containing anthracyclines, taxanes, platinum and PARPi; ATPtPD1, containing anthracyclines, taxanes, platinum and PD-1 inhibitor; TPt, containing taxanes and platinum.

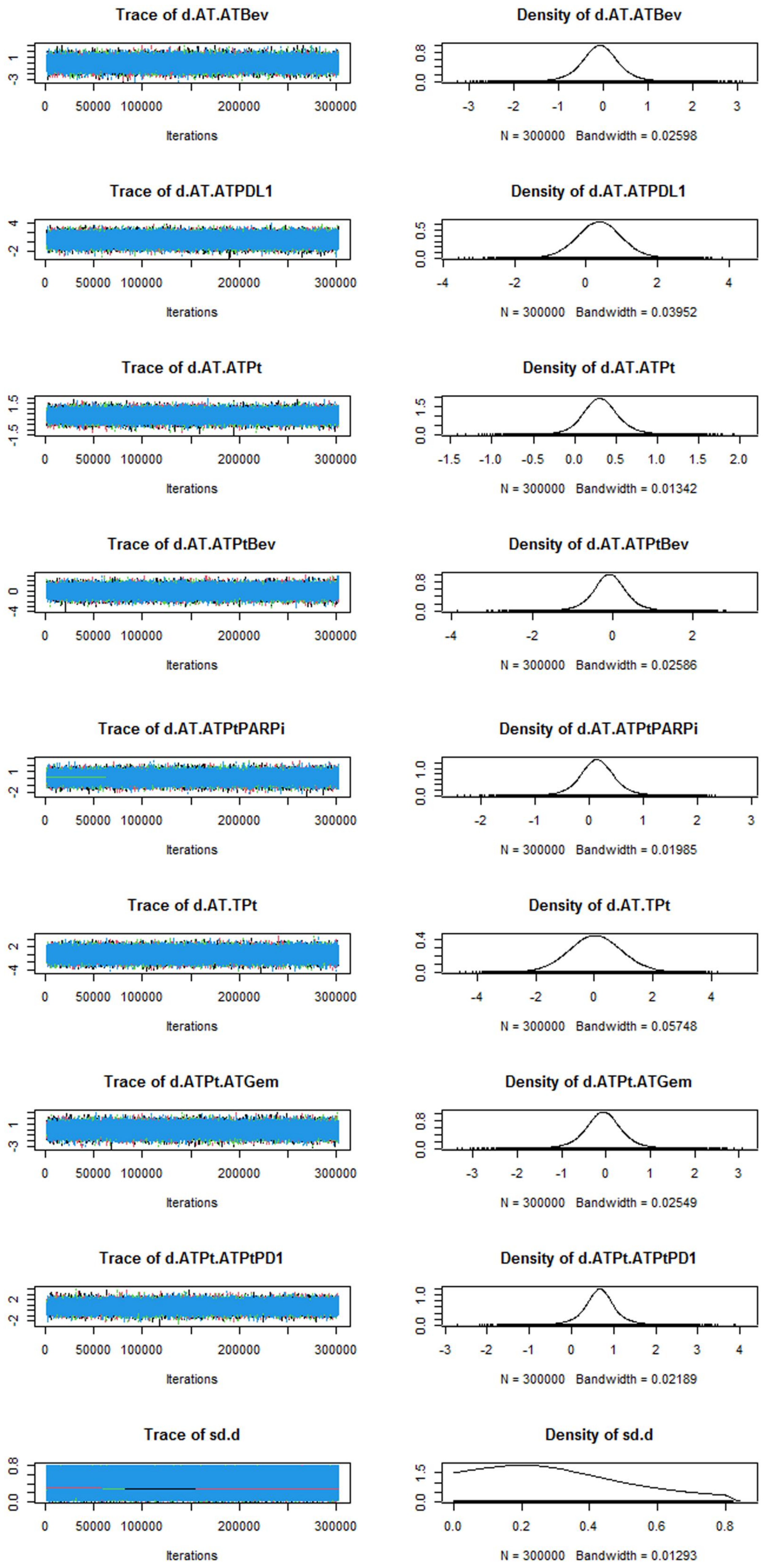

Figure S5. Convergence of the three Markov Chain Monte Carlo chains established by of the history feature for HR of overall survival after regression analysis. AT, containing anthracyclines and taxanes; ATBev, containing anthracyclines, taxanes and bevacizumab; ATGem, containing anthracyclines, taxanes and gemcitabine; ATPDL1, containing anthracyclines, taxanes and PD-L1 inhibitor; ATPt, containing anthracyclines, taxanes and platinum; ATPtBev, containing anthracyclines, taxanes, platinum and bevacizumab; ATPtPARPi, containing anthracyclines, taxanes, platinum and PARPi; ATPtPD1, containing anthracyclines, taxanes, platinum and PD-1 inhibitor; TPt, containing taxanes and platinum.

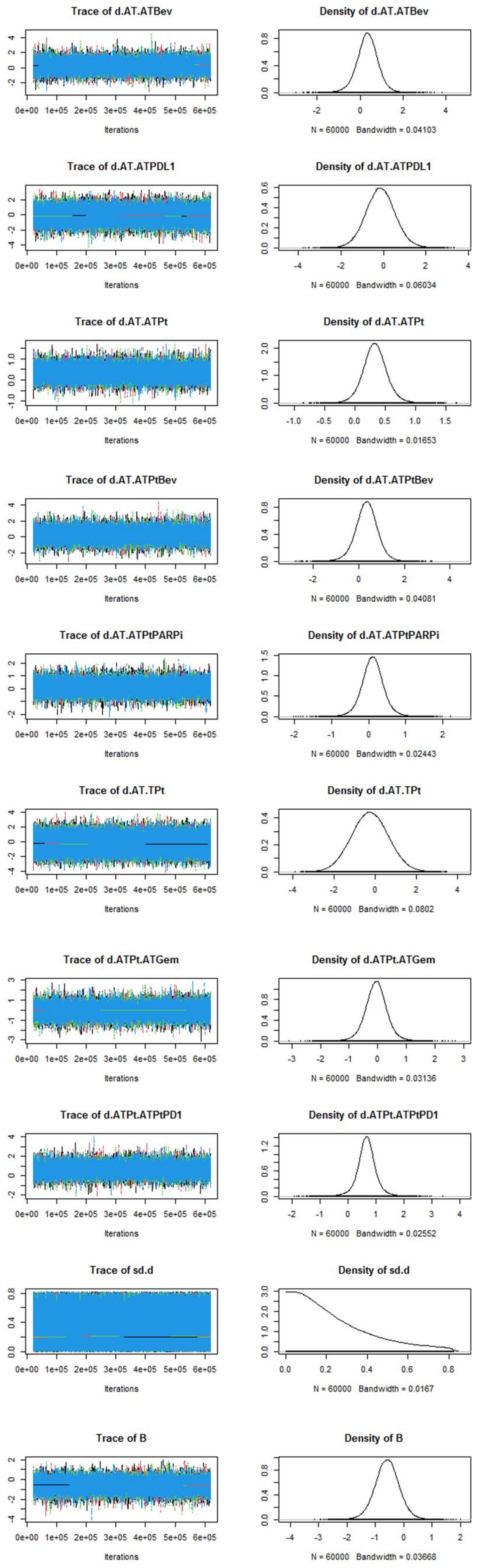

Figure S6. Convergence of the three Markov Chain Monte Carlo chains established by of the Brooks-Gelman-Rubin diagnostic for HR of events free survival. AT, containing anthracyclines and taxanes; ATBev, containing anthracyclines, taxanes and bevacizumab; ATGem, containing anthracyclines, taxanes and gemcitabine; ATPDL1, containing anthracyclines, taxanes and PD-L1 inhibitor; ATPt, containing anthracyclines, taxanes and platinum; ATPtBev, containing anthracyclines, taxanes, platinum and bevacizumab; ATPtPARPi, containing anthracyclines, taxanes, platinum and PARPi; ATPtPD1, containing anthracyclines, taxanes, platinum and PD-1 inhibitor; TPt, containing taxanes and platinum; TPtEve, containing taxanes, platinum and everolimus.

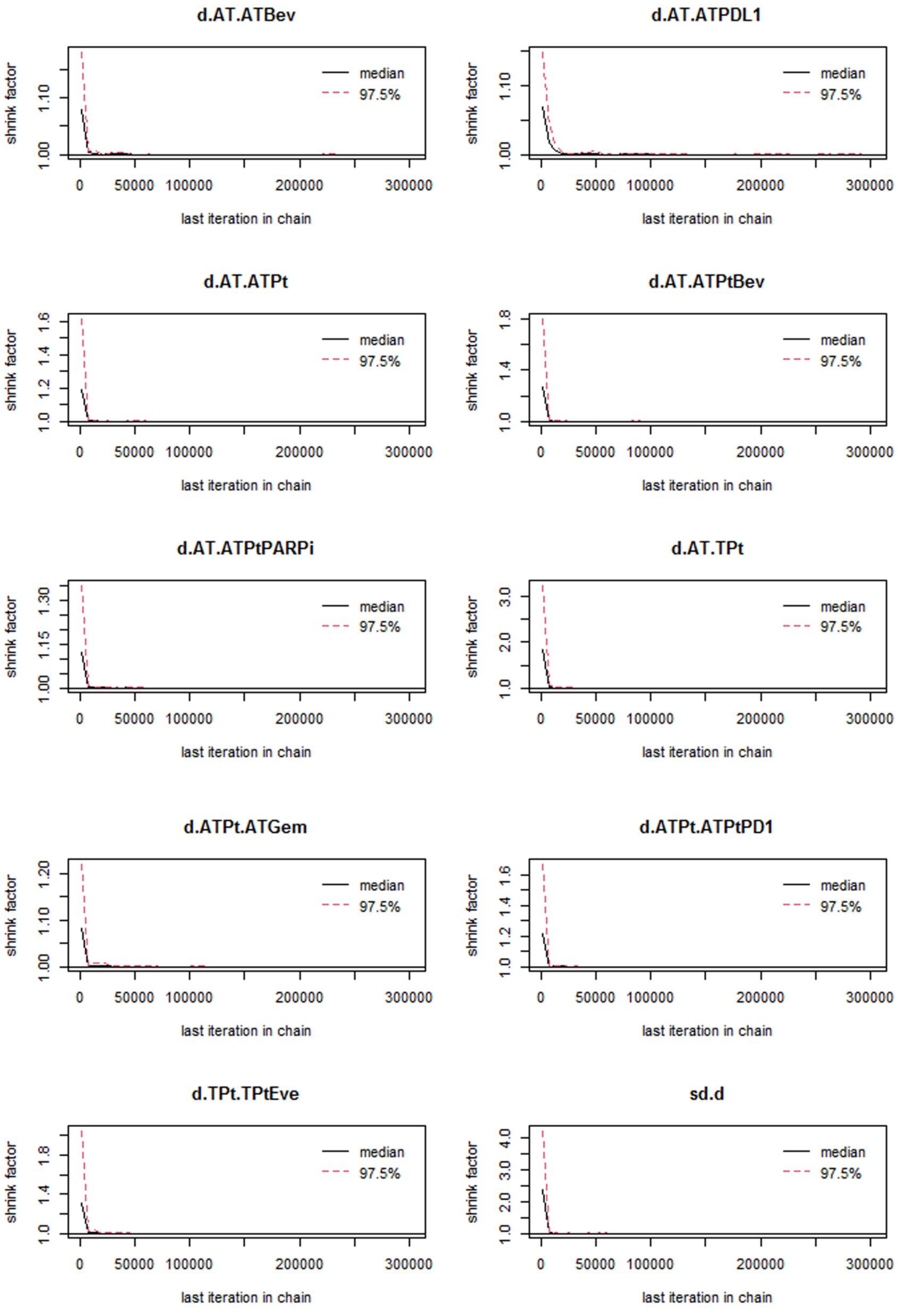

Figure S7. Convergence of the three Markov Chain Monte Carlo chains established by of the Brooks-Gelman-Rubin diagnostic for HR of events free survival after regression analysis. AT, containing anthracyclines and taxanes; ATBev, containing anthracyclines, taxanes and bevacizumab; ATGem, containing anthracyclines, taxanes and gemcitabine; ATPDL1, containing anthracyclines, taxanes and PD-L1 inhibitor; ATPt, containing anthracyclines, taxanes and platinum; ATPtBev, containing anthracyclines, taxanes, platinum and bevacizumab; ATPtPARPi, containing anthracyclines, taxanes, platinum and PARPi; ATPtPD1, containing anthracyclines, taxanes, platinum and PD-1 inhibitor; TPt, containing taxanes and platinum; TPtEve, containing taxanes, platinum and everolimus.

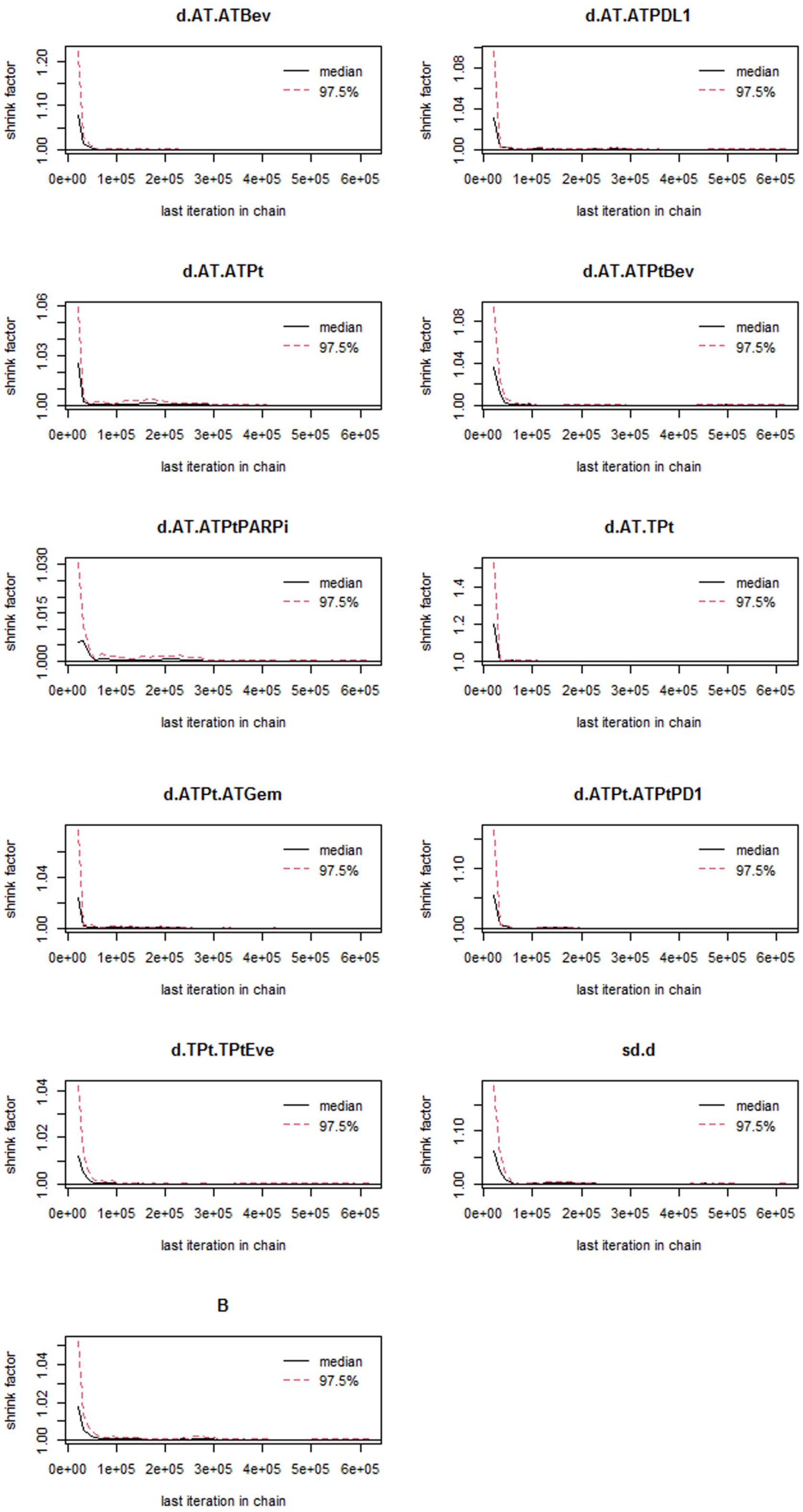

Figure S8. Convergence of the three Markov Chain Monte Carlo chains established by of the Brooks-Gelman-Rubin diagnostic for HR of overall survival. AT, containing anthracyclines and taxanes; ATBev, containing anthracyclines, taxanes and bevacizumab; ATGem, containing anthracyclines, taxanes and gemcitabine; ATPDL1, containing anthracyclines, taxanes and PD-L1 inhibitor; ATPt, containing anthracyclines, taxanes and platinum; ATPtBev, containing anthracyclines, taxanes, platinum and bevacizumab; ATPtPARPi, containing anthracyclines, taxanes, platinum and PARPi; ATPtPD1, containing anthracyclines, taxanes, platinum and PD-1 inhibitor; TPt, containing taxanes and platinum.

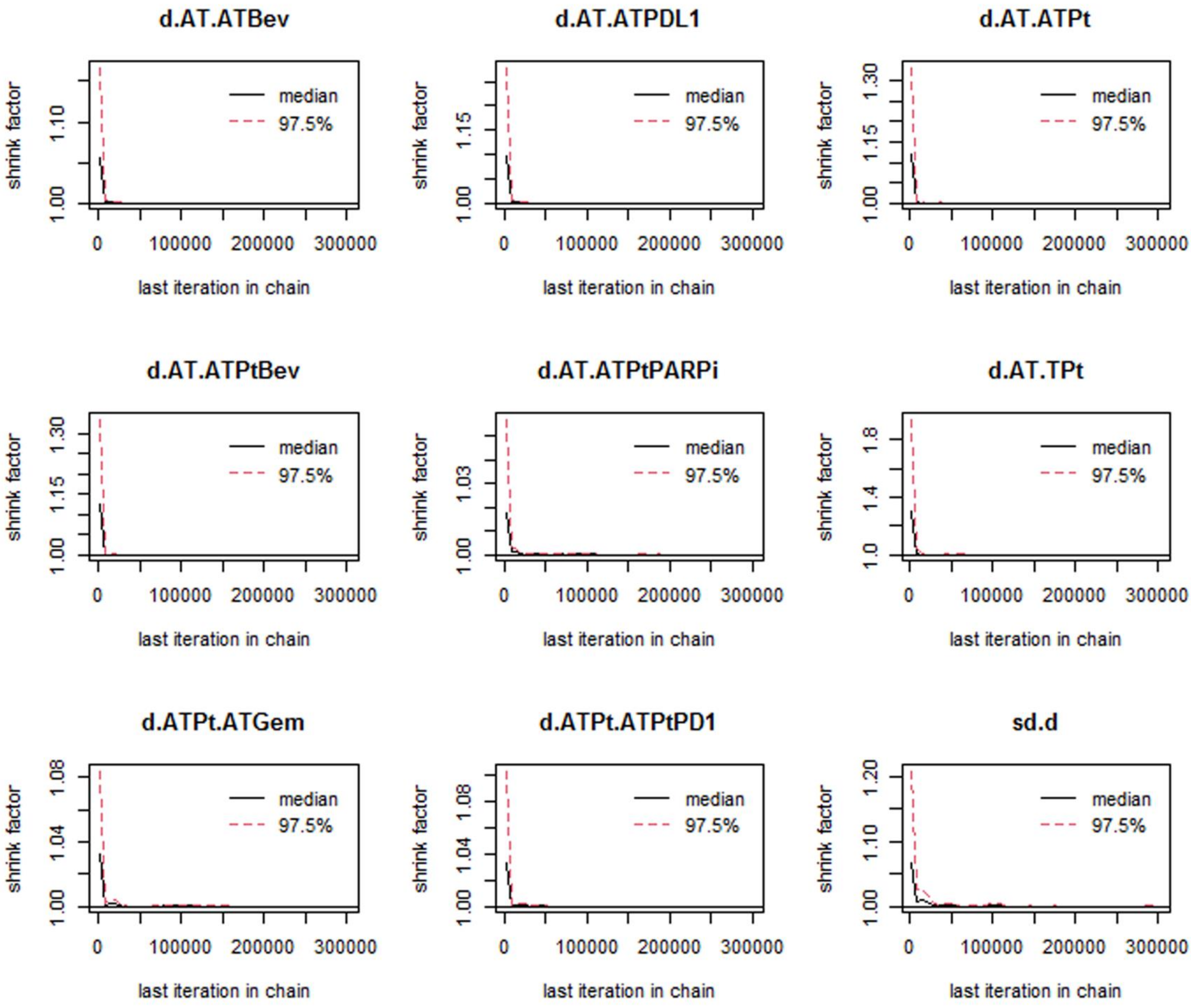

Figure S9. Convergence of the three Markov Chain Monte Carlo chains established by of the Brooks-Gelman-Rubin diagnostic for HR of overall survival after regression analysis. AT, containing anthracyclines and taxanes; ATBev, containing anthracyclines, taxanes and bevacizumab; ATGem, containing anthracyclines, taxanes and gemcitabine; ATPDL1, containing anthracyclines, taxanes and PD-L1 inhibitor; ATPt, containing anthracyclines, taxanes and platinum; ATPtBev, containing anthracyclines, taxanes, platinum and bevacizumab; ATPtPARPi, containing anthracyclines, taxanes, platinum and PARPi; ATPtPD1, containing anthracyclines, taxanes, platinum and PD-1 inhibitor; TPt, containing taxanes and platinum.

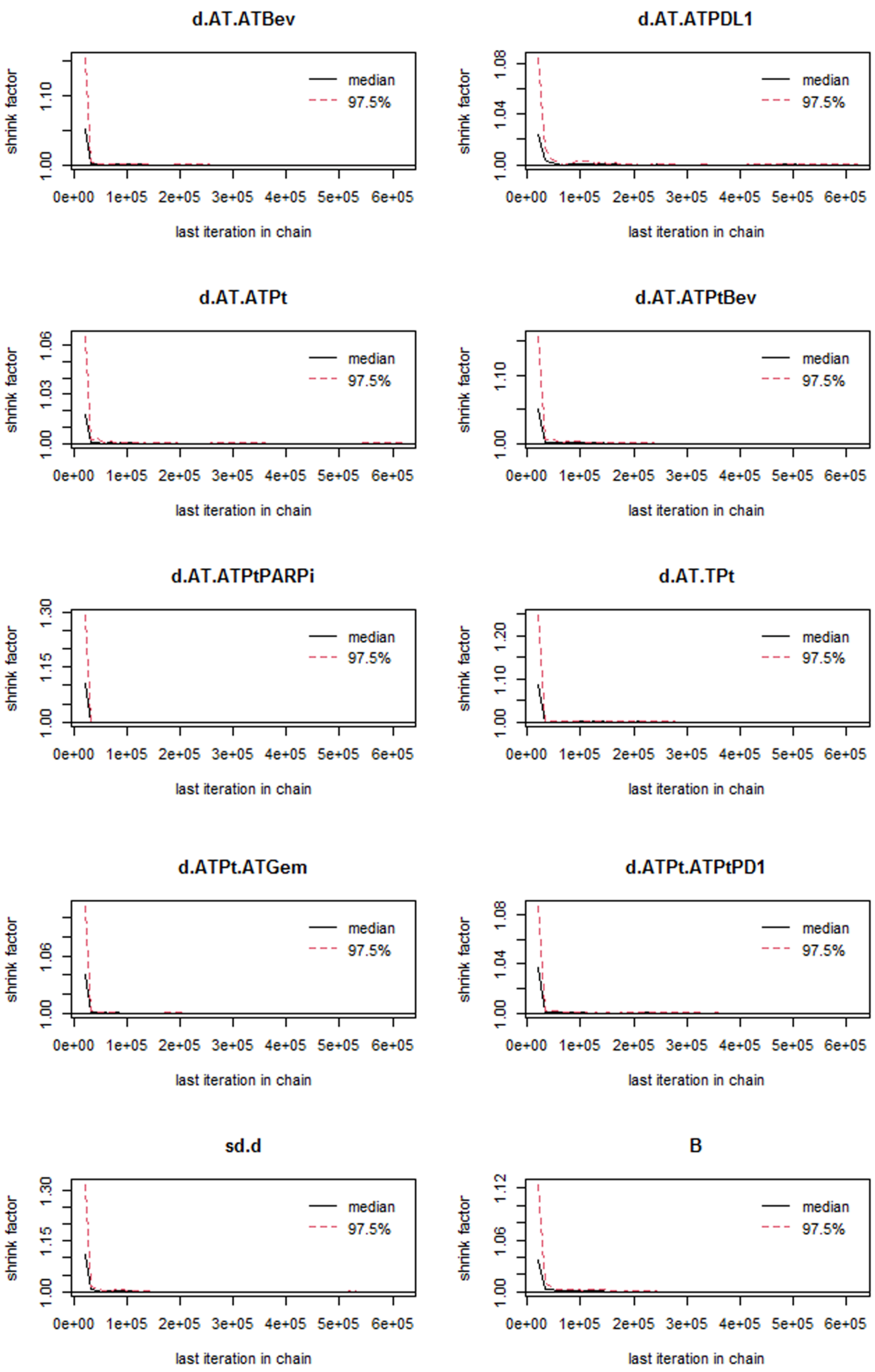

Figure S10. Network funnel plot of pairwise comparisons of PCR rate. PCR, pathological complete response; AT, containing anthracyclines and taxanes; ATBev, containing anthracyclines, taxanes and bevacizumab; ATEve, containing anthracyclines, taxanes and everolimus; ATGem, containing anthracyclines, taxanes and gemcitabine; ATPD1, containing anthracyclines, taxanes and PD-1 inhibitor; ATPDL1, containing anthracyclines, taxanes and PD-L1 inhibitor; ATPt, containing anthracyclines, taxanes and platinum; ATPtBev, containing anthracyclines, taxanes, platinum and bevacizumab; ATPtPARPi, containing anthracyclines, taxanes, platinum and PARPi; ATPtPD1, containing anthracyclines, taxanes, platinum and PD-1 inhibitor; ATPtPDL1, containing anthracyclines, taxanes, platinum and PD-L1 inhibitor; ATX, containing anthracyclines, taxanes and capacitabine; T, containing taxanes only(T); TPARPi, containing taxanes and PARPi; TPt, containing taxanes and platinum; TPtEve, containing taxanes, platinum and everolimus; TPtPDL1, containing taxanes, platinum and PD-L1 inhibitor.

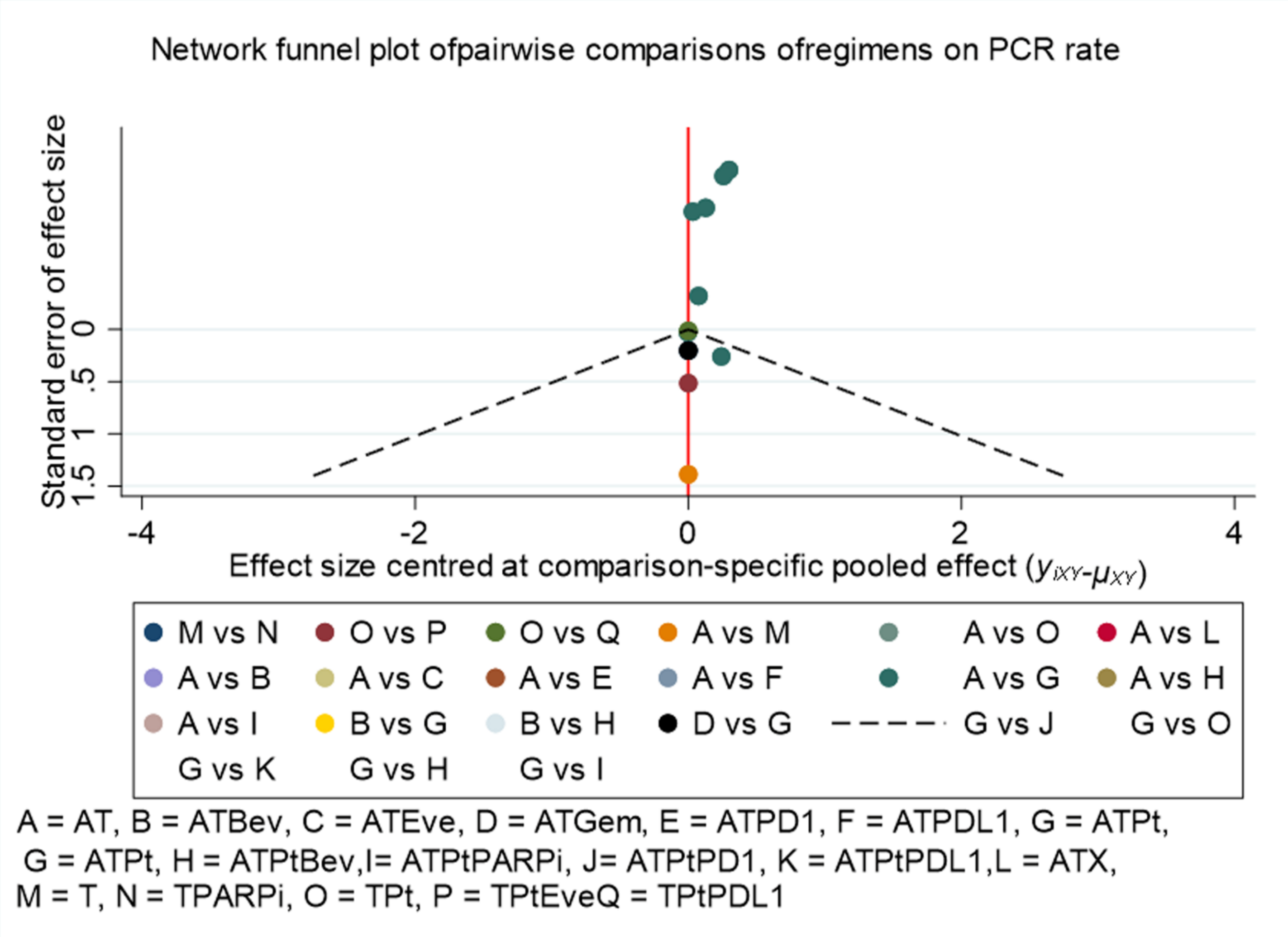

Figure S11. Network funnel plot of pairwise comparisons of each time point of EFS. EFS, events free survival; AT, containing anthracyclines and taxanes; ATGem, containing anthracyclines, taxanes and gemcitabine; ATPD1, containing anthracyclines, taxanes and PD-1 inhibitor; ATPt, containing anthracyclines, taxanes and platinum; ATPtPD1, containing anthracyclines, taxanes, platinum and PD-1 inhibitor; T, containing taxanes only(T); TPt, containing taxanes and platinum; TPtEve, containing taxanes, platinum and everolimus.

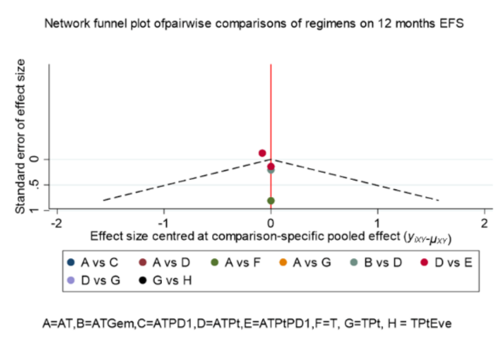

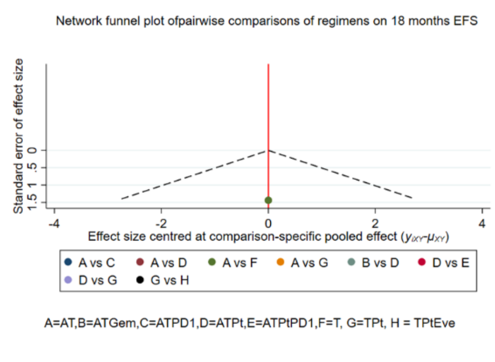

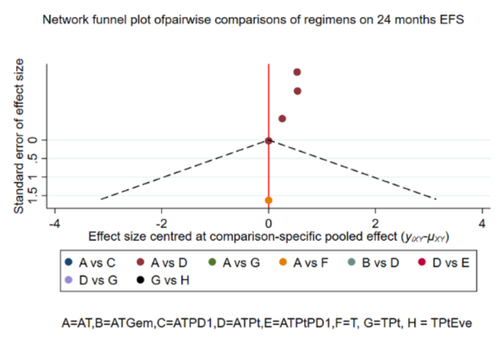

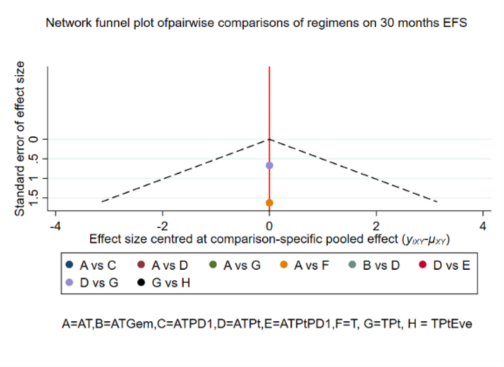

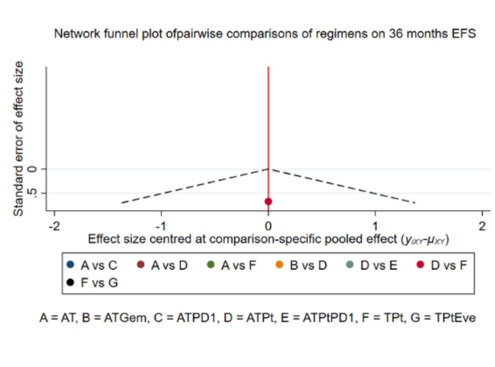

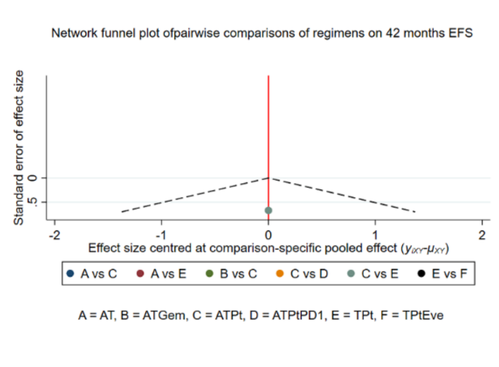

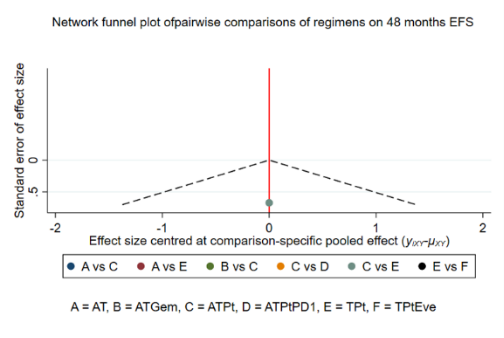

Figure S12. Network funnel plot of pairwise comparisons of each time point of OS. OS, overall survival; AT, containing anthracyclines and taxanes; ATGem, containing anthracyclines, taxanes and gemcitabine; ATPt, containing anthracyclines, taxanes and platinum; ATPtPD1, containing anthracyclines, taxanes, platinum and PD-1 inhibitor; TPt, containing taxanes and platinum; T, containing taxanes only.

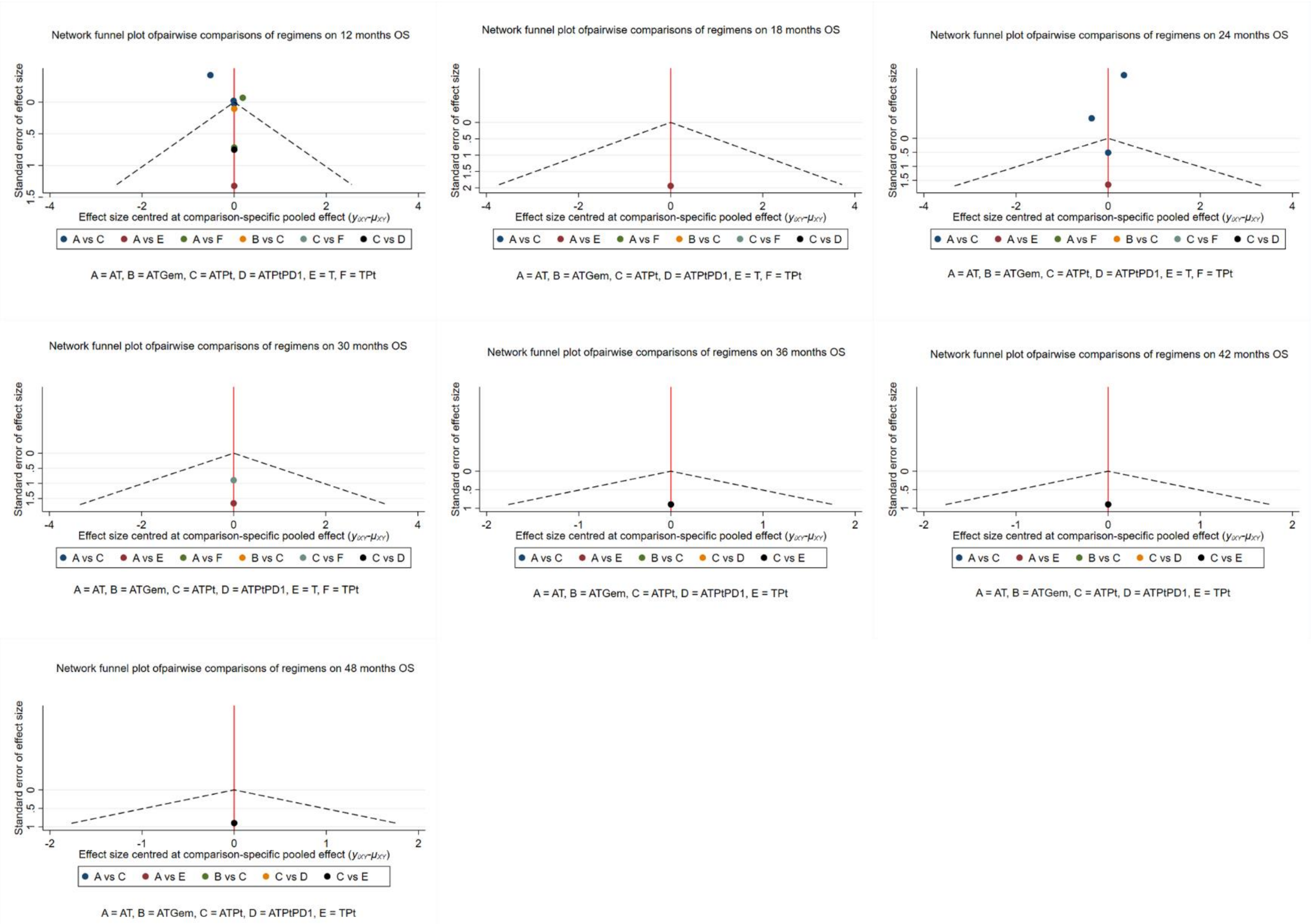

Supplement: Supplementary file 1 [file medi-105-e46962-s001.pdf]
